# Complex plant quality—microbiota–population interactions modulate the response of a specialist herbivore to the defence of its host plant

**DOI:** 10.1111/1365-2435.14177

**Published:** 2022-09-15

**Authors:** Guillaume Minard, Aapo Kahilainen, Arjen Biere, Hannu Pakkanen, Johanna Mappes, Marjo Saastamoinen

**Affiliations:** ^1^ Organismal and Evolutionary Biology Research Programme University of Helsinki Helsinki Finland; ^2^ Université de Lyon Lyon France; ^3^ Ecologie Microbienne UMR CNRS 5557, UMR INRA 1418, VetAgro Sup, Université Lyon 1 Villeurbanne France; ^4^ Finnish Environment Institute Biodiversity Centre Helsinki Finland; ^5^ Department of Terrestrial Ecology Netherlands Institute of Ecology (NIOO‐KNAW) Wageningen The Netherlands; ^6^ Department of Chemistry University of Jyväskylä Jyväskylä Finland; ^7^ Department of Biological and Environmental Science University of Jyväskylä Jyväskylä Finland; ^8^ Helsinki Institute of Life Sciences University of Helsinki Helsinki Finland

**Keywords:** herbivore, Lepidoptera, microbiota, plant defence, trophic interactions

## Abstract

1. Many specialist herbivores have evolved strategies to cope with plant defences, with gut microbiota potentially participating to such adaptations.
2. In this study, we assessed whether the history of plant use (population origin) and microbiota may interact with plant defence adaptation.
3. We tested whether microbiota enhance the performance of *Melitaea cinxia* larvae on their host plant, *Plantago lanceolata* and increase their ability to cope the defensive compounds, iridoid glycosides (IGs).
4. The gut microbiota were significantly affected by both larval population origin and host plant IG level. Contrary to our prediction, impoverishing the microbiota with antibiotic treatment did not reduce larval performance.
5. As expected for this specialized insect herbivore, sequestration of one of IGs was higher in larvae fed with plants producing higher concentration of IGs. These larvae also showed metabolic signature of intoxication (i.e. decrease in Lysine levels). However, intoxication on highly defended plants was only observed when larvae with a history of poorly defended plants were simultaneously treated with antibiotics.
6. Our results suggest that both adaptation and microbiota contribute to the metabolic response of herbivores to plant defence though complex interactions.

Read the free Plain Language Summary for this article on the Journal blog.

## Graphical abstract

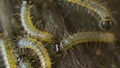

## Résumé

1. De nombreux herbivores spécialistes ont évolué vers des stratégies qui leurs permettent de contourner les défenses de leur plantes hôtes. Le microbiote pourrait potentiellement participer à certaines de ces adaptations.
2. Dans cette étude, nous avons essayé de déterminer si l'adaptation d'un herbivore est influencée par son microbiote et l'historique d'utilisation de sa plante hôte (origine de la population).
3. Nous avons testé en quoi le microbiote contribue à la performance de chenilles *Melitaea cinxia* sur leur plante hôte *Plantago lanceolata* ainsi que leur capacité à faire face aux glucosides d'iridoïdes (GI), des molécules de défenses produites par *P. lanceolata*.
4. Comme attendu, la concentration de GI stockée était plus importante chez les chenilles qui étaient nourries avec des plantes produisant de fortes concentrations de GI. Ces chenilles présentaient par ailleurs des signes d'intoxications (i.e. diminution de la concentration de Lysine). Cependant cela n'était visible que lorsque les chenilles étaient issues de populations qui se nourrissaient historiquement sur des plantes peu défendues et lorsqu'elles étaient simultanément traitées par des antibiotiques.
5. Nos résultats suggèrent donc que des processus complexes d'adaptation couplés à l'activité du microbiote contribuent à la réponse des herbivores aux défenses de leurs plante hôtes.

## 1 INTRODUCTION

In most ecosystems, communities are regulated through a network of trophic interactions (Fretwell, 1987). In these networks, herbivore–plant interactions play a central role and often drastically impact the dynamics of the rest of the community (e.g. bottom‐up interactions with predators, parasites or top‐down interactions with detritivores), as well as matter fluxes and nutrient cycles (Fretwell, 1987; Metcalfe et al., 2014). Both partners of this interaction are involved in a coevolutionary arms race, in which the plant evolves new traits against herbivores, while in turn, herbivores adapt to these traits to be able to consume the plant (Ehrlich & Raven, 1964). Coevolutionary theory predicts that such forces lead to the diversification of both partners, and symmetric diversification patterns have been observed between plants and herbivores across many empirical data (Futuyma & Agrawal, 2009).

Being sessile, plants are easily exposed to herbivory. Instead, plants have developed an array of chemical and physical traits as defence mechanisms against herbivores that act either constitutively or after induction [reviewed by Aljbory & Chen (2018)]. Many plants produce secondary metabolites that aim to reduce the performance of the herbivores through direct (repellent, toxins) or indirect defence (attract another trophic level such as a predator or a parasitoid of the herbivore). Among compounds involved in direct defence, terpenoids are by far the most diverse and ubiquitous class with more than 30,000 different molecules (Mithöfer & Boland, 2012). They are followed by alkaloids (~12,000 molecules) and phenolic compounds (~9000 molecules). The functions of defensive metabolites are diverse and often poorly characterized. As an example, some of them alter the digestion, induce oxidative stress and cytotoxicity (Bhonwong et al., 2009; Treutter, 2005).

Due to their common dual antiherbivory and antimicrobial activity, recent studies have suggested that in certain cases, the main target of defensive compounds may not be the herbivore itself but instead its gut microbiota (Hammer & Bowers, 2015). Modification of the herbivore's microbiota could then negatively impact the physiology and consequently the performance of the herbivore feeding on the defended host plant (Hammer & Bowers, 2015; Mithöfer & Boland, 2012). Conversely, the gut microbiota may also be involved in the detoxification of defensive compounds, and thus contribute to the adaptation of specialist herbivores to highly defended host plants (Hammer & Bowers, 2015). For example, the microbiota of pine weevils and bark beetles help their host to degrade pine tissues rich in terpenoid resins or phenolic compounds (Berasategui et al., 2017; Cheng et al., 2018). These coleopterans can, thus, only interact efficiently with their host plant when they form a holobiont composed of the insect host and specialized microorganisms.

Similar patterns may also be evident in Lepidoptera. Recent studies have, however, suggested that Lepidoptera harbour a transient and highly variable microbiota with limited impact on host performance and primary metabolism (Duplouy et al., 2020; Hammer et al., 2017; Whitaker et al., 2016). Conversely, few studies have indicated that this relatively transient microbiota can contribute to lepidopteran immunity (Duplouy et al., 2020; Galarza et al., 2021; Mason et al., 2019; Yoon et al., 2019), that some microbes might lead to transgenerational responses and facilitate host plant shifts (Voirol et al., 2020), or in contrast elicit host plant defences (Wang et al., 2017). Interactions regarding potential detoxification of plant defensive compounds, however, remain poorly studied.

In order to further understand the potential relevance of host plant defence–herbivore–microbiota interactions, we used a well‐characterized plant–herbivore study system represented by the host plant ribwort plantain (*Plantago lanceolata*) and its specialist herbivore, the Glanville fritillary (*Melitaea cinxia*) butterfly. The bioactive metabolites of *Plantago lanceolata* have previously been investigated (Tamura & Nishibe, 2002) and *P. lanceolata* is known to synthesize aucubin and catalpol, two monoterpenoids that belong to the class of iridoid glycosides (IGs) that carry antibacterial, antifungal and antipredatory properties (Baden & Dobler, 2009; Davini et al., 1986; Marak et al., 2002; Reudler et al., 2011).

The molecules are not active while formed in the plant and are compartmentalized within the vacuole. Whenever a herbivore or a pathogen degrades the plant cell, the plant will release the IGs that could then react with the β‐glucosidases of either the plant, the herbivore or the pathogen to form reactive aglycones (Kim et al., 2000; Pankoke et al., 2013). In another system, it has been demonstrated that the aglycone links proteins through an activated dialdehyde that forms protein adducts and reacts with the side chain of lysine, an essential amino acid (Mander & Liu, 2010). This reaction decreases the amount of lysine available, denatures digestive enzymes and decreases the nutritional value of the food to herbivores and pathogens. The same dialdehyde structure appears after activation of aucubin by β‐glucosidases and consistently results in decreased levels of lysine (Kim et al., 2000; Konno et al., 1999).

Some specialist herbivores have, however, adapted to cope with iridoid glycosides, and even store them for their own defence against parasites and predators (Bowers & Puttick, 1986). Previous studies in *M. cinxia* have shown that pre‐diapause larvae contain between 0.2% and 2% of IGs, mostly catalpol (Nieminen et al., 2003; Suomi et al., 2001, 2003). All studies so far have been assessing an entire individual, and thus, it remains unclear whether larvae actively transport and store the IG compounds in specific tissues or whether the compounds are maintained within the gut. However, as 1‐day starved larvae as well as adult butterflies (which do not feed anymore on the host plant) contain equivalent levels of IGs, it has been suggested that the compounds are actively sequestered (Nieminen et al., 2003; Suomi et al., 2003). This may contribute to the higher tolerance of larvae to their natural enemies when they are feeding on highly defended plants (Laurentz et al., 2012; Nieminen et al., 2003; Reudler et al., 2011). Studies assessing the impacts of high IG levels in host plants on larval performance vary between no effects to a small increase in weight and development rate (Laurentz et al., 2012; Reudler et al., 2011; Saastamoinen et al., 2007). In addition to IGs, *P. lanceolata* also produce the high levels of a phenolic compound, the phenylethanoid glycoside verbascoside (alternatively named acteoside) (Tamura & Nishibe, 2002), which antimicrobial and antiherbivory properties remain controversial (Fazly Bazzaz et al., 2018; Holeski et al., 2013; Pardo et al., 1993; Reichardt et al., 1988).

In this study, our first objective was to investigate whether the microbiota of *M. cinxia* affect the performance of larvae. We selected prediapause larvae since they live as gregarious families on a single host plant (contrarily to postdiapausing larvae) and their condition partially explains their survival probability during diapause (Duplouy et al., 2018; Kahilainen et al., 2018; Kuussaari & Singer, 2017; Tack et al., 2015). Our second objective was to evaluate whether any differences in performance are mediated by effects of the microbiota on the ability of the host larvae to cope with the defences of their host plants. Our third objective was to assess whether the composition of the larval gut microbiome and its effect on larval performance differ among larvae whose parents originated from populations with a different history of host plant use.

In order to investigate the impact of larval microbiota on larval performance and the ability of larvae to cope with IGs, we addressed the following three questions: (1) Do microbiota confer an advantage to their host larvae? For this, we used leaf antibiotic treatments to manipulate the larval microbiome and tested whether larvae feeding on untreated leaves had higher performance (survival, development rate, biomass production) than larvae feeding on antibiotics‐treated leaves. (2) Can any advantages conferred by microbiota be related to mitigation of the impact of ingested host IGs? For this, we used selection lines of *P. lanceolata*, in which plants had been selected for high or low constitutive levels of leaf IGs (high‐IG and low‐IG lines) (Marak et al., 2000). We assessed the metabolomes (including levels of IGs and lysine) of larvae fed on antibiotics‐treated and untreated leaves of high‐IG and low‐IG plants to test (i) whether larvae fed high‐IG lines had higher levels of IGs and reduced levels of lysine, (ii) whether microbiota mitigated a reduction in lysine levels and (iii) whether larvae feeding on untreated high‐IG plants had higher performance than larvae of antibiotics‐treated high‐IG plants. (3) Does the parental origin of the larvae affect the microbiome composition of the larvae and its effect on larval performance and ability to cope with IGs? For this, we used *M. cinxia* larvae whose parents originated from populations in two distinct regions (Eckerö and Sund) within the Åland archipelago in Finland that differ in their evolutionary history of host use. Populations from Eckerö have a history of encountering lower defence levels due to the presence of two host plant species, *Veronica spicata* and *Plantago lanceolata*, that generally contain low and high levels of IGs respectively. By contrast, larvae originating from Sund have a history of encountering only the more highly defended host plant *P. lanceolata*. We studied the composition of their microbiota and performance after being exposed to contrasted IGs concentrations. We thus specifically tested (i) whether the assembled gut bacterial and fungal microbiome of larvae differs between parental population origin and (ii) whether larvae originating from regions with a stronger evolutionary history with high‐IG plants (Sund) benefit more from their associated microbiome when feeding on high‐IG plants than larvae from regions with a weaker evolutionary history with high‐IG plants (Eckerö).

## 2 MATERIALS AND METHODS

### 2.1 Plant selection lines and rearing

**FIGURE 1:**
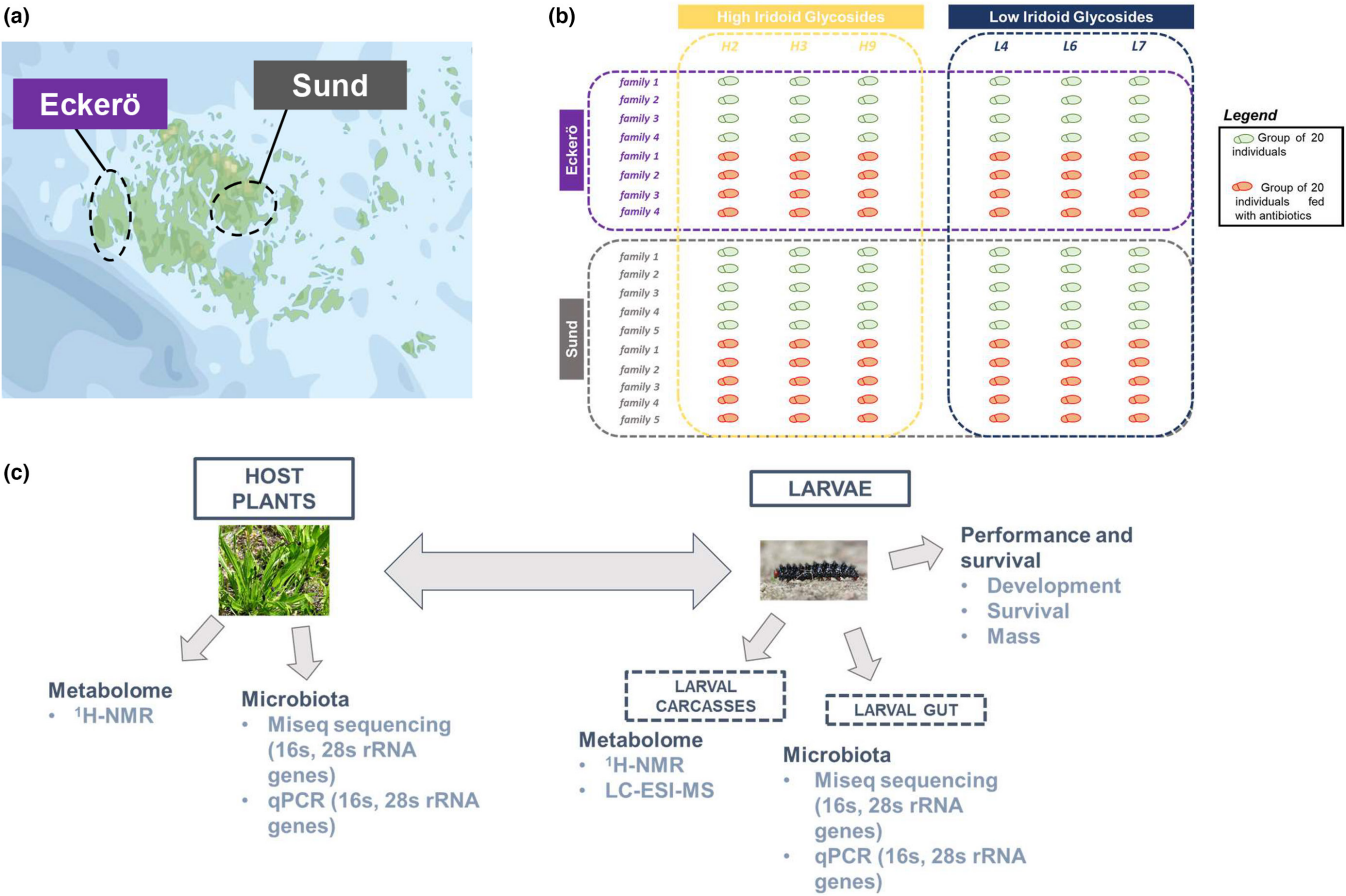
Experimental design. (a) Larval families were obtained from parents collected as larvae in Åland islands. Males and females that were mated came from different habitat patches belonging to different population networks. Five and four families were obtained from individuals collected in Eckerö and Sund, respectively. (b) Each larvae represent a group of 20 individuals that were daily fed with a leaf of *Plantago lanceolata* collected from a plant genotype selected to produce either high (H2, H3, H9) or low (L4, L6, L7) levels of plant defence (iridoid glycosides or IGs). (c) The larval performance and survivorship of larvae were recorded. Prokaryotic and eukaryotic microbiota of plant leaves and larval gut were investigated through metabarcoding and qPCR. The metabolism linked to plant defence and the insect response was investigated through ^1^H‐NMR and LC‐ESI MS

The seeds of the *P. lanceolata* selection lines were obtained from the Netherlands Institute of Ecology (NIOO‐KNAW). The two lines of plants have been selected to produce either high or low concentrations of Iridoid Glycosides (IG), as previously described (Marak et al., 2000). A total of six genotypes (three from each line) were selected from a pool of 20 genotypes (see Supplementary methods). The leaves of these plants were used to feed the larvae during a period of 27 days according to the experimental design described below (Figure 1). To track any variation that may have occurred during the experiment, samples from the host plants were randomly harvested from six plants every 3 days, immediately freeze‐dried and stored at −80°C for metabolomics analysis.

### 2.2 Experimental design and measurements

Parental lines of the butterflies were sampled in the spring of 2016 at the end of larval diapause (fifth and sixth instar larvae) in two distinct regions (Eckerö and Sund) located in two large islands of the Åland archipelago, Finland. No permit was required to collect this species. Within each region, the individuals were collected from three habitat patches belonging to the same semi‐independent habitat network (Hanski et al., 2017). Larvae from the two regions have previously been shown to be genetically differentiated (Nair et al., 2016) and they also show ecological differences: habitats from Eckerö are colonized by two host plant species of the butterfly, *P. lanceolata* and *Veronica spicata*, of which the butterfly prefers *V. spicata* in these regions. The habitats from Sund contain only *P. lanceolata* and the butterflies also slightly prefer this species when both host plants are provided in a choice test (Kuussaari et al., 2000). It is important to note that the two plant species produce the same IG compounds, but IG levels in *V. spicata* are more than twofold lower than in *P. lanceolata* (Saastamoinen et al., 2007). For the present experiment, five females from Sund and four females from Eckerö were mated with a male collected from the same region but from a different local population (in order to avoid inbreeding). Females were allowed to lay eggs and the larvae hatching from these eggs (females lay clutches of eggs) were used for the feeding assay: A full‐factorial design in which a group of 20 individuals from each family was assigned to each treatment (see Figure 1 for a scheme of the experimental design). In total, 2160 larvae were used (6 plant families × 9 herbivore families × 2 antibiotics treatments × 20 larvae). In order to avoid clutch effects, larvae from different clutches were randomly distributed across the treatments. Each group was fed with leaves from one of the six selected plant genotypes that were either supplemented with 200 μl of water (Control) or 200 μl of an antibiotic solution (Antibiotic) described below. The plant leaves were harvested, rinsed with water and a piece of 1.7 cm^2^ of plant leaves was deposited on a sterile petri dish containing 20 larvae. Leaves from the same plant genotype were collected from at least three individual plants to provide food to the larvae and the same plant was not used in two successive days in order to limit the enhancement of plant defensive response. Water or antibiotic solutions were deposited at the surface of the plant leaf piece. This procedure was executed every day for each petri dish. The antibiotic solution contained three antibacterial and two antifungal compounds and was prepared according to the previously published method with some modifications (Chung et al., 2013). The composition of the antibiotic solution was 2 × 10^−4^ g ml^−1^ of neomycin sulphate, 1 × 10^−3^ g ml^−1^ aureomycin and 6 × 10^−5^ g ml^−1^ streptomycin and 8 × 10^−4^ g ml^−1^ methyl paraben, 6 × 10^−4^ g ml^−1^ sorbic acid. As we were able to divide each initial larval family (eggs from a single female) into twelve 20 larvae groups, each family was fed with all of the six selected genotypes of *P. lanceolata* with or without antibiotics (i.e. split‐family design). Different egg clutches were used for this analysis. The development and survival of the larvae to the second instar was recorded in order to characterize larval life‐history traits. The larvae that survived and reached the third instar were sampled, snap frozen and stored at −80°C. The mass of a group of three third instar larvae per family per treatment was recorded, and the rest of the larvae were used for metabarcoding, qPCR and metabolomics analysis. The larvae were reared in groups and group size was recorded as it is known to impact larval performance in the gregarious phases of the larval development (Saastamoinen, 2007).

All the samples were prepared under a flow hood with sterile material and proper protection in order to limit contaminations. The larvae were dissected in 1X PBS (GIBCO) on a platform refrigerated with ice <0°C to limit IG conversions. Dissected guts were stored in 1X PBS and carcasses (including the dissection buffer) were stored without any additional buffer. Dissected body parts were immediately frozen in liquid nitrogen and stored at −80°C.

Guts of three *M. cinxia* individuals per family, plant genotype and treatment were pooled. Those pools were used to identify the microbial communities colonizing each conditions through bacterial and fungal metabarcoding approaches and qPCR quantifications (see details in *Supplementary methods*). In addition, three 0.5‐cm^2^ leaf pieces per plant genotype were analysed with the same approach.

The metabolome of the larvae and the plant, carcasses from the pooled individuals were analysed with ESI‐MS and ^1^H‐NMR. Metabolome from 3.5 mg of freeze‐dried plant leaves from each genotype was analysed with ^1^H‐NMR (see details in Supplementary methods).

The experimental design and measurements are summarized in Figure 1.

### 2.3 Statistical analysis

All analyses were computed using R (R Core Team, 2016). Factors significantly impacting the plant metabolites and the larval microbiota or metabolites were tested with a permutational multivariate analysis of variance (*adonis‐*ANOVA) with the package vegan (Oksanen et al., 2013). The relative concentrations of metabolites were scaled and centred prior to the analyses in order to limit the impact of highly abundant metabolites. Global variations in the plant metabolite composition across plant IG selection lines (‘Plant line’), plant genotypes within selection lines (‘Plant genotype’) or time was conducted through redundancy analysis (RDA). Differences in the specific plant metabolites between plant lines, plant genotypes and time were assessed using a linear model (LM). OTUs explaining the most part of the variation in the larval microbiota were represented through principal coordinate analysis (PCoA) based on Bray–Curtis dissimilarities in community composition between samples. Microbial and metabolite composition across larvae were represented with bar plots and heat maps. Comparisons of metabolites and microorganisms present in larval gut samples across the two sites of parental origin of the larvae (‘Larval origin’), the two plant lines and the two antibiotic treatments were conducted with the package lme4 (Bates et al., 2017, p. 4) by computing linear mixed models (LMM) with two random effects (i) plant genotype nested within plant line and (ii) larval family nested within larval origin. The impact of covariates (i.e. the two sites of parental origin of the larvae, the two plant lines and the two antibiotic treatments) on larval life‐history traits was compared by using an LMM (mass) or a generalized linear mixed model (GLMM) with a Poisson distribution and a log‐link function (development time) or a binomial distribution and a probit link function (survival). Plant genotype nested within plant line and larval family nested within larval origin were used as random effect. Tukey post hoc tests were conducted to test for differences between levels with the emmeans package (Lenth et al., 2022).

## 3 RESULTS

### 3.1 Do microbiota confer an advantage to their host larvae?

#### 3.1.1 Broad‐spectrum antibiotic treatment induces an erosion of the microbiota towards specific taxa

**FIGURE 2:**
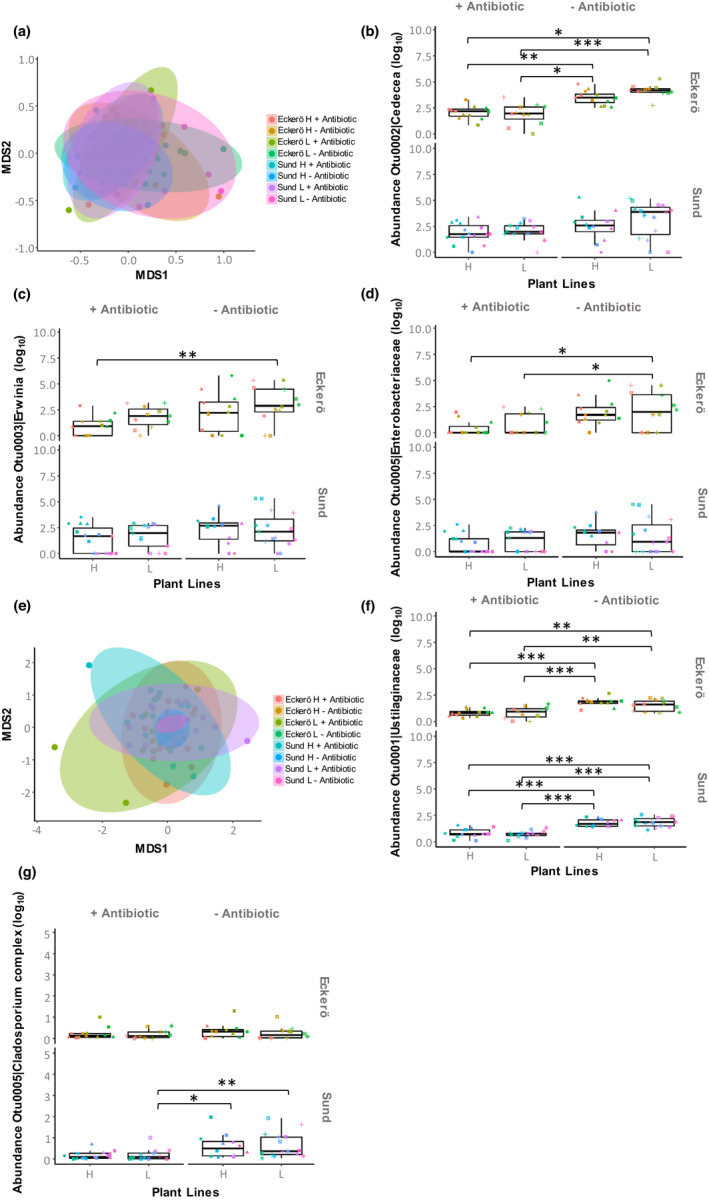
Variation in microbiota. (a) The nonmetric multidimensional scaling represents similarities (Bray–Curtis distances) in the bacterial communities across treatment groups. Abundance data are shown for the three most important bacterial OTUs namely (b) *Cedecea* sp.—Otu0002, (c) *Erwinia* sp.—Otu0003 and (d) an unclassified Enterobacteriaceae—Otu0005. (e) The non‐metric multidimensional scaling represents similarities (Bray–Curtis distances) in the fungal communities across treatment groups. Abundance data are shown for the two most important bacterial OTUs namely (f) an unclassified Ustilaginaceae—Otu0001, (g) *Cladosporium* sp.—Otu0005. Plant selection lines that contain high‐IG are referred as ‘H’ while those containing low‐IG are referred as ‘L’. Each dot represents a pool of three individuals from the same petri dish. Different point shapes represent pools of individuals fed with the same plant genotype. Different point colours represent pools of individuals that come from different families. Statistical significances from Tukey post hoc tests are reported for *p* < 0.05 ‘*’, *p* < 0.01 ‘**’ and *p* < 0.001 ‘***’

The bacterial and fungal gut communities of the larvae were mainly affected by the antibiotic treatment (Table 1). The fungal community being, however, more affected than the bacterial community with a drastic reduction of the community similarity variation after treatment (*R* ^2^ = 0.086 and 0.237, respectively, for bacteria and fungi; Figure 2a,e; Figure S3). When considering the OTU levels, bacterial and fungal communities were mostly influenced by the variation in the abundance of three and two OTUs, respectively, that were also the most abundant taxa retrieved from the larval gut (Figure S2). These OTUs were identified as *Cedecea* sp. (Otu0002), *Erwinia* sp. (Otu0003) and an unclassified Enterobacteriaceae (Otu0005) for the bacteria, and as Ustilaginaceae unclassified (Otu0001) and *Cladosporium* complex (Otu0005) for the fungi. Reduction in the density of these dominant bacterial OTUs between antibiotic treated and non‐treated larvae was only observed for larvae originating from the region Eckerö within the archipelago, while larvae from both regions (Eckerö and Sund) experienced reduction in the density of the most dominant fungal OTUs (Figure 2b–g; Table S2).

**TABLE 1:** Analysis of the factors influencing the global composition of the larval microbiota

Community^a^	Factors	df	Pseudo‐*F*	*R* ^2^	*p*
Bacteria	Origin	1	2.586	0.023	0.003**
	Category	1	2.369	0.021	0.009**
	Treatment	1	9.767	0.086	0.001***
	Origin × Category	1	1.192	0.011	0.202
	Origin × Treatment	1	1.370	0.121	0.111
	Category × Treatment	1	1.711	0.150	0.042*
	Origin × Category × Treatment	1	0.762	0.007	0.719
	Residuals	94		0.826	
Fungi	Origin	1	0.349	0.003	0.901
	Category	1	0.186	0.001	0.992
	Treatment	1	31.670	0.237	0.001***
	Origin × Category	1	0.504	0.004	0.757
	Origin × Treatment	1	1.324	0.010	0.226
	Category × Treatment	1	1.104	0.008	0.305
	Origin × Category × Treatment	1	1.572	0.012	0.173
	Residuals	94		0.725	

^a^ Bray–Curtis dissimilarity distances.

. *p* ≤ 0.1, *p ≤ 0.05, **p ≤ 0.01, ***p ≤ 0.001.

#### 3.1.2 Larvae show little differences in performance and survivorship

**FIGURE 3:**
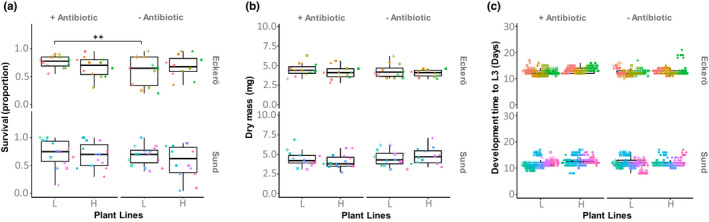
Survival and performance of the larvae across the treatment groups. (a) The survival rate, (b) the dry mass and (c) the development time to L3 have been measured either on average for the whole population (survival) for pools of three individuals (dry mass) or individually (development time). Plant selection lines that contain high‐IG are referred as ‘H’ while those containing low‐IG are referred as ‘L’. Different point shapes represent individuals fed with the same plant genotype. Different point colours represent individuals that comes from different families. Statistical significances from Tukey post hoc tests are reported for *p* < 0.01 ‘**’

Larvae fed with antibiotic did not consistently show divergence in their development time and mass (*p* = 0.340 and 0.427, respectively, see details Table 2) in comparison with non‐treated larvae. Only survival was significantly impacted by the treatment with a slight increase in survival of individuals from Eckerö fed with low‐IG plant and antibiotics (Figure 3a).

**TABLE 2:** Factors influencing the larvae larval performance and survivorship

Variable	Factor^a^	*df*	*χ* ^2^	*p*
Development to L3	Plant lines (high‐IG vs. low‐IG)	1	7.612	0.006**
	Antibiotic treatment	1	0.911	0.340
	Larval origin (Sund vs. Eckerö)	1	2.329	0.126
	P. lines × Ab. treatment	1	2.127	0.145
	P. lines × Larv. origin	1	0.202	0.652
	Ab. treatment × Larv. origin	1	0.008	0.927
	P. lines × Ab. treatment × Larv. origin	1	0.333	0.564
Survival to L3	Plant lines (high‐IG vs. low‐IG)	1	0.516	0.473
	Antibiotic treatment	1	13.257	0.0003***
	Larval origin (Sund vs. Eckerö)	1	0.067	0.796
	P. lines × Ab. treatment	1	1.431	0.232
	P. lines × Larv. origin	1	2.633	0.105
	Ab. treatment × Larv. origin	1	0.070	0.792
	P. lines × Ab. treatment × Larv. origin	1	8.383	0.004**

		*df*	F	*p*
Mass (L3 larvae)	Plant lines (high‐IG vs. low‐IG)	1,4.074	0.573	0.491
	Antibiotic treatment	1,84.846	0.637	0.427
	Larval origin (Sund vs. Eckerö)	1,7.357	1.549	0.251
	P. lines × Ab. treatment	1,85.035	1.054	0.308
	P. lines × Larv. origin	1,84.933	0.654	0.421
	Ab. treatment × Larv. origin	1,84.933	2.379	0.127
	P. lines × Ab. treatment x Larv. origin	1,85.126	0.505	0.479

^a^ Larval families nested by origin and plant genotype nested by category were used as random factors.

* *p* ≤ 0.05, **p ≤ 0.01, ***p ≤ 0.001.

The larval body mass (estimated after collection at L3) was not impacted by the antibiotic treatment or by the IG selection line category of the host plant they were fed with (Table 2, Figure 3b). Larvae originating from the different regions showed no difference in survival, development time (until L3) or body mass. The development time of larvae fed with high‐IG lines was significantly longer (Table 2), but this difference was smaller than one day on average (12.8 ± 0.28 days for the High‐IG lines and 12.0 ± 0.26 for the low‐IG lines) and contrast Tukey tests (that are more conservative) did not evidence no significant differences in development among individuals belonging to both regions (Figure 3c).

### 3.2 Can any advantages conferred by microbiota be related to mitigation of the impact of ingested host IGs?

#### 3.2.1 IG selection leads to broad plant metabolome variations

Overall, selection for high‐IG and low‐IG explained 27% of the metabolite variation among the plants (Table 3; Figure S4). In addition, the composition of the plant metabolites varied among the plant genotypes and changed over time (Table 3; Figure S4). The two defensive compounds (aucubin, catalpol) targeted by the selection process were confirmed to be lower in low‐IG lines compared to high‐IG lines (difference for aucubin relative concentration: CI 95% = −0.045 to −0.020; *F*_1,50_ = 31.97; *p* = 7.4 × 10^−7^; catalpol: CI 95% = −0.055 to −0.033; *F*_1,50_ = 84.94; *p* = 2.33 × 10^−12^). A different defensive compound (i.e. verbascoside) showed also variation in response to the selection treatment but was not regarded in this study (Figure S4).

**TABLE 3:** Factors influencing the variations of the plant metabolites

Factor	df	Pseudo *F*	*R* ^2^	*p*
Plant line (high‐IG vs. low‐IG)	1	31.04	0.272	0.001***
Time	1	11.18	0.098	0.001***
Plant genotype (nested in plant line)	4	3.45	0.121	0.002**
Plant line × Time	1	0.43	0.004	0.767
Plant genotype × Time (nested in plant line)	4	1.88	0.066	0.035*
Residuals	50		0.439	

* *p* ≤ 0.05, **p ≤ 0.01, *** ≤ 0.001.

#### 3.2.2 Larvae from different origins show a signature of protein alteration after being fed with high levels of iridoid glycosides and this effect is accentuated in the presence of microbiota

**FIGURE 4:**
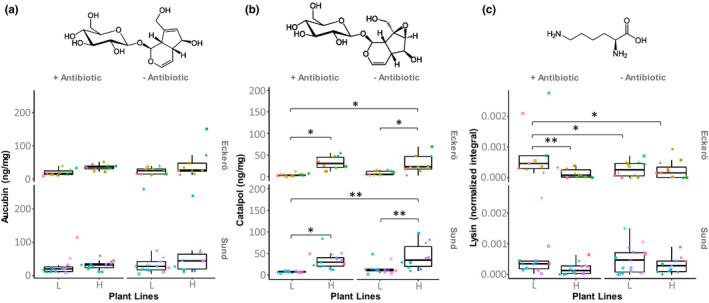
Iridoid glycosides and lysine variations in larvae across the treatment groups. (a) Aucubin and (b) catalpol were measured with LC‐ESI‐MS while (c) lysine was measured with ^1^H‐NMR across the treatment group. Plant selection lines that contain high‐IG are referred as ‘H’ while those containing low‐IG are referred as ‘L’. Different point shapes represent pools of individuals fed with the same plant genotype. Different point colours represent pools of individuals that come from different families. Statistical significances from Tukey post hoc tests are reported for *p* < 0.05 ‘*’ and *p* < 0.01 ‘**’

Comparisons of the metabolite composition in larvae was conducted on pooled carcasses of three individuals after gut removal. Generally, levels of aucubin and catalpol stored within larval carcasses were too low to be efficiently assessed through ^1^H‐NMR spectra (Figure S5). Therefore, these analyses were performed using LC‐ESI‐MS. The results showed that larvae stored low concentrations of IGs (36.56 ± 39.90 and 21.97 ± 20.89 ng.mg^−1^ or 10^−4^% for aucubin and catalpol, respectively). These values can, however, be slightly underestimated since the larvae were dissected on ice, and thus, some metabolite conversion may have occurred resulting in decreased IG concentration in the carcasses. None of the studied factors significantly impacted the aucubin concentration within larval carcasses (Table 4), whereas larvae that had fed on high‐IG lines stored more catalpol than those that had fed on low‐IG lines (Figure 4a,b). This result suggests that the larval storage of IG in the early developmental instars is weak but depends on the concentration of these compounds in the host plant.

**TABLE 4:** Analysis of factors influencing the concentration of compounds potentially involved in iridoid glycoside metabolism in larvae

Compound	Factors^a^	df	Den df	*F*	*p*‐value
Lysine	Larval origin (Sund vs. Eckerö)	1	7.134	0.0018	0.967
	Plant lines (high‐IG vs. low‐IG)	1	85.554	12.1480	7.778 × 10^−4^***
	Antibiotic treatment	1	85.551	1.2888	0.259
	Larv. origin × P. lines	1	85.554	0.7781	0.380
	Larv. origin × Ab. treatment	1	85.551	2.9272	0.091
	P. lines × Ab. treatment	1	86.059	5.7047	0.019*
	Larv. origin × P. lines × Ab. treatment	1	86.059	2.7729	0.100
Aucubin	Larval origin (Sund vs. Eckerö)	1	7.071	0.504	0.501
	Plant lines (high‐IG vs. low‐IG)	1	3.783	2.961	0.165
	Antibiotic treatment	1	69.227	1.044	0.311
	Larv. origin × P. lines	1	70.021	0.789	0.378
	Larv. origin × Ab. treatment	1	67.754	0.564	0.455
	P. lines × Ab. treatment	1	69.719	0.013	0.911
	Larv. origin × P. lines × Ab. treatment	1	67.930	0.173	0.679
Catalpol	Larval origin (Sund vs. Eckerö)	1	7.263	2.909	0.130
	Plant lines (high‐IG vs. low‐IG)	1	3.501	38.951	0.005**
	Antibiotic treatment	1	71.039	1.194	0.278
	Larv. origin × P. lines	1	70.963	0.037	0.848
	Larv. origin × Ab. treatment	1	67.639	0.663	0.418
	P. lines × Ab. treatment	1	71.448	0.003	0.954
	Larv. origin × P. lines × Ab. treatment	1	67.862	0.535	0.467

^a^ Larval families nested by larval origin and plant genotype nested by plant lines were used as random factors.

* *p* ≤ 0.05, **p ≤ 0.01, ***p ≤ 0.001.

The activation of IGs consists of a deglycosilation, which leads to the release of glucose and a reactive aglycone that binds lysine residues. We found that the detectable levels of lysine were lower in larvae from Eckerö fed with leaves from high‐IG lines compared to those fed with leaves from low‐IG lines (Table 4; Figure 4c). These results suggest that the iridoid glycosides are at least partially activated in the larvae and that they efficiently reduce the amount of accessible lysine within the individuals. In addition, there was an interaction between plant IG line and the antibiotic treatment; individuals form Eckerö that had been fed on low‐IG line host plants harboured a higher amount of lysine when their microbiota were altered by antibiotics compared with untreated individuals (Figure 4c), while no difference was observed between antibiotic treated and untreated individuals fed with plants producing high levels of IGs. This suggests either that the microbiota contribute to the activation of the IGs in those larvae or that part of the lysine acquired through the food is used by the microbiota of the larvae. It is noteworthy, however, that we cannot exclude the potential direct effect of the antibiotic treatment. Interestingly, this effect was only observed in the larvae originating from Eckerö region that has a history of poorly defended plant consumption (i.e. *Veronica spicata*) but not on the larvae from Sund that naturally feed on more highly defended plants (i.e. *Plantago lanceolata*).

### 3.3 Does the origin of the larvae affect their microbiome composition and its effect on larval performance and ability to cope with IGs?

#### 3.3.1 Larval gut microbiome is altered by antibiotic treatment and is only mildly affected by host plant defensive compounds and larval origin

The microbiota of the plant leaves, assessed by analysing leaves from three individual plants per genotype, were dominated by the bacterial taxa *Cedecea*, unclassified Enterobacteriaceae and *Cutibacterium* (Figure S6). The bacterial microbiota of the plant differed between IG selection lines (pseudo‐*F*_1,12_ = 0.184, *p* = 0.005; Table S3), whereas the fungal communities of the plant leaves were not influenced by IG selection line (pseudo‐*F*_1,12_ = 0.921, *R* ^2^ = 0.054, *p* = 0.514). The microbiota of the non‐antibiotic treated larvae strongly differed from that of its host plant (pseudo‐*F*_1,68_ = 14.77, *R* ^2^ = 0.17, *p* = 0.004 and pseudo‐*F*_1,67_ = 30.27, *R* ^2^ = 0.31, *p* = 0.001 for bacteria and fungi, respectively; Figure S3). In addition to the effects of antibiotics, the bacterial microbiota communities were also impacted by the origin of the larvae and by the plant IG selection line they were fed on (Table 1), but these effects were weaker. The fungal community was not impacted by the origin of the larvae or by the IG selection line (of the host plant).

When considering the OTU levels, the dominant bacterial and fungal taxa were differentially impacted by the antibiotic treatment according to the region of their origin and the host plant treatment. As an example, the Otu0005 of the *Cladosporium* complex decreases after antibiotic treatment in larvae fed with the low‐IGhost plants and only for individuals originating from Sund. Otu0002 assigned to *Cedecea* decreases in larvae fed with either low and high‐IG plants but only when larvae originated from Eckerö regions (Figure 2). Such complex microbiota × host population x plant lines interactions might partially explain why microbiota mediated decrease of survivorship and mitigation of the larval metabolism in response to IG was only observed for populations from Eckerö but not that from Sund.

## 4 DISCUSSION

In some higher eukaryotes, microbiome plays an important role in nutrient intake and a range of life‐history traits. However, there are still contradictory views about how important microbiome is for vital functions in Lepidoptera that seem to lack the permanent microbiome. In this study, we asked whether the microbiota of *M. cinxia* could influence its larval performance before winter diapause and if plant defences intercede with those potential responses. We also assessed whether the microbiota and their effect on the larval performance vary between populations of different history of host plant use.

Three main conclusions can be derived from this study. (1) The reduction of the larval microbiome following antibiotic treatment of the ingested food slightly increases the survival of the larvae but only when they originate from regions with a history of more poorly defended plant use and when fed on plants producing low levels of IG. (2) In larvae from same region only, the larval microbiome mitigated the reduction in lysine levels that occurred when larvae were feeding on a high‐IG diet as compared to a low‐IG diet. (3) Despite the fact that lepidopteran larval microbiomes have previously been suggested to be transiently acquired from the environment, the origin of the larvae had a significant effect on the microbiota even when the larvae themselves had been reared in common‐garden, and this difference influenced their ability to cope with IGs. Below, we discuss these results according to the biological questions that were addressed.

### 4.1 Do microbiota confer an advantage to their host larvae?

#### 4.1.1 Altering the microbiota with an antibiotic treatment increases the survival of *M. cinxia* pre‐diapausing larvae but only under certain circumstances

In our study, key developmental traits were not affected by the disturbance of the larval microbiota. Survival was even slightly higher in larvae from Eckerö when fed with antibiotics. Therefore, we conclude that insect‐associated microbes are not mandatory for larval performance even when the host plant quality changes and that microorganisms might even be slightly costly for their insect host. In a previous study on the same insect model, antibiotic treatment incurred a slight cost towards larval development and survival although it did not affect the metabolism of larvae (Duplouy et al., 2020). The complex interactions between population origin of the larvae and the defence levels of the host plants fed might explain the difference between these studies, although the results converged in the sense that either of them evidenced high amplitude changes in the traits tested. Our result is also consistent with studies on the tobacco hornworm, which showed that growth and survival was not altered when the insect microbiota were disturbed through antibiotic treatments (Hammer et al., 2017). In the tiger moth, antibiotic treatment even lead to a faster development of individuals, which may be attributed to a relaxation of the immunity that is often costly (Dickel et al., 2016). The latter study, however, revealed negative consequences on later traits (i.e. lower fecundity), which we did not investigate here. Most recently, disruption and restoration of the microbiota of two Nymphalidae butterflies (*Danaus chrysippus and Ariadne merione*) also failed to demonstrate any consequences of the microbiota for survival and development of caterpillars (Phalnikar et al., 2019). It is, however, noteworthy to highlight that larvae with a history of low‐IG plant use did experience higher survival when fed on low‐IG plants after antibiotic treatment. Therefore, the interaction between *M. cinxia* and its microbiota might vary depending on the plant defence levels and evolutionary history of the populations considered.

### 4.2 Can the microbiota be related to mitigation of the impact of ingested host IGs?

#### 4.2.1 Sequestration of catalpol by *M. cinxia* larvae depends on larval origin and plant IG concentration

The signals of Aucubin and Catalpol were poorly detectable in the larvae compared to their detectability in the host plants. Consequently, it seems that at least in the early developmental instars, these compounds are poorly sequestered by the larvae. IGs sequestration is a strategy used by some specialist and generalist herbivores to hijack the plant defence for their own protection against predators and parasites (Baden & Dobler, 2009; Bowers & Puttick, 1986; Lampert & Bowers, 2010). Our experiment evidenced that once the gut is removed, the stored concentration of IGs is ~100 times lower (i.e. 36.56 ± 39.90 × 10^−4^ and 21.97 ± 20.89 × 10^−4^% for Aucubin and Catalpol, respectively) than what was measured from the entire individuals (Laurentz et al., 2012; Nieminen et al., 2003; Reudler Talsma et al., 2008; Suomi et al., 2001, 2003), with concentrations of Catalpol in the larvae correlating with the concentration of this same molecule in the host plant. However, even with weak active sequestration at these early instars, we cannot exclude that the IGs contained in the gut or the carcass are sufficient in quantity to deter herbivores or parasites. Furthermore, active storage of IGs might be enhanced only before and during specific stages when the individuals are not actively feeding on the plant to compensate for the fact that they do not carry plant IGs within their gut (e.g. in diapausing larvae, pupae and adults). Interestingly, larvae that were fed with high‐IG lines showed a signature of iridoid glycoside activation through a significant reduction of lysine. Thus, contrary to what would be expected for a specialist herbivore, the IGs do seem to impact the larval metabolism. This was only observed in larvae from Eckerö and for antibiotic treated larvae, and was also correlated with the survival rate of the individuals. We could thus speculate that IG activation does alter the survival of the larvae. However, further investigation is required to assess whether and why those modifications impact the insect physiology. The common buckeye butterfly *Junonia coenia* is a specialist herbivore whose larvae can develop on *P. lanceolata* leaves and store IGs. A previous study demonstrated that, when fed with high IG food, the concentration of compounds stored by larvae was negatively correlated with their growth rate (Camara, 1997). The authors suggested that IGs might be costly for the butterfly but still provide a net fitness benefit under conditions where individuals have to defend themselves against natural enemies. Other studies have also reported that cost–benefit relationship between iridoid sequestration and predator defence in generalist herbivores depends on the environmental context (Lindstedt et al., 2010; Reudler et al., 2015). A previous study investigating the impact of the plant selection lines on pre‐diapause larvae (Saastamoinen et al., 2007) showed that such costs associated with IGs concentration might vary across developmental stages (i.e. larvae developed slower while feeding on high‐IG lines during the first instar but then exhibited a faster development during the second and third instar). Another recent study showed that larvae fed with high‐IG lines tend to grow faster until diapause (Rosa et al., 2018). This can be explained by the fact that larvae fed with high IG leaves consume larger amounts of leaves or have higher efficiency of conversion of leaf biomass into caterpillar biomass at later stages (Saastamoinen et al., 2007). It is also important to note that major variation in family response to the host plant quality was previously reported and can mitigate the global trend at higher levels (Kahilainen et al., 2021).

#### 4.2.2 Larval microbiota mitigate the impact of IG on the metabolism when IGs are ingested at high concentration

According to predictions, our results showed that larvae fed with high‐IG plant lines experienced a Lysin reduction compared to larvae fed with low‐IG plant lines due to the irreversible crosslinking of dietary protein, notably lysine, by IG aglycones. However, this was only observed for antibiotic treated larvae from Eckerö. The decrease in lysine concentration in the presence of the microbiota might be due to a direct competition between the larvae and their hosted microbes for this amino acid and/or an increase of IG activation in the presence of microorganisms (which secrete their own β‐glucosidases). In *Drosophila melanogaster*, the absence of microbiota leads to an increase in glucose and triacylglyceride, which are reduced, if the flies are recolonized by any of their five dominant bacterial symbionts (Newell & Douglas, 2014). Several examples have shown that the links between Lepidopteran gut microbiota and plant defences are complex. Some microbes contribute directly to detoxification. *Hyphantria cunea* is one of the most invasive agricultural and forest pest, and resistant to cinnamic acids, a defence compound that induces oxidative stress in insects. Increases of bacteria subsequent to the exposition of individuals to that compound correlate with the production of enzymatic and non‐enzymatic antioxidant as well as detoxification enzymes (Jiang et al., 2021). An example of intoxication is a study by Chen et al. (2021) that showed how DMNT, a plant defence volatile, damages the peritrophic matrix of *Plutella xylostella* and alters the composition of its microbiota. Peritrophic matrix is a physical barrier that isolates food and protects the insect gut from pathogen invasion. The authors showed that the microbiota are mandatory for DMNT‐induced killing of the larvae. Mortality, in this case, was most likely due to opportunistic pathogen infecting the insect. Negative interactions between microbiota and plan defences might also be indirect as in the polyphagous moth *Helicoverpa zea* that carries a gut bacteria, *Enterobacter ludwigii*, which enhances the production of protein glucose oxidase in the insect saliva (Wang et al., 2018). This enzyme acts as an elicitor that stimulates the defence of maize plants colonized by *H. zea*. The microbiota of Lepidopteran larvae may also induce various responses depending on the host plant species. Saliva‐associated bacteria in the fall armyworm, *Spodoptera frugiperda*, induces modulations of plant defences that confer an advantage to the insect on tomato but a disadvantage on maize (Acevedo et al., 2017). In our study, we evidenced that the microbiota may contribute to the mitigation of IG toxicity without no influence on its sequestration.

### 4.3 Does the parental origin of the larvae affect their microbiome composition and influence larval performance and ability to cope with IGs?

#### 4.3.1 Parental origin of larvae affects the larval microbiome of a lepidopteran species

Larvae originating from parents collected from two different areas of the Åland islands showed small but significant differentiation in their microbiota composition even when reared under controlled conditions. This pattern was especially notable in regard to the bacteria *Cedecea* sp. and the fungi *Cladosporium* sp. This might result from differences in evolutionary or co‐evolutionary histories between the two insect populations or epigenetic environmentally induced parental effects that might have been persisted in the population after they were transferred to the laboratory. Such a pattern is often observed when symbionts are vertically transmitted or when environmentally acquired symbionts are selected by specific expression of the host genotype (Hernández‐Martínez et al., 2010; Lee et al., 2016; Näpflin & Schmid‐Hempel, 2016). In particular, variations in the host immune receptor or effectors could be important as they have already been proven to be highly interconnected with the caterpillar's microbial communities (Duplouy et al., 2020; Rosa et al., 2019). Even though under laboratory conditions, the origin of the larvae can predict their colonization by a given microorganism, further studies are necessary to confirm whether similar patterns can be observed in the field. The colonization depends on the identity of the microbes it interacts with but also on the probability of acquisition and maintenance of the microorganism due to experienced environmental conditions. A previous investigation conducted on 121 lepidopteran species suggested that they mostly acquire transient microbes that are poorly related to the environment or the insect genotype (Hammer et al., 2017). Other studies highlighted the importance of soil as a key reservoir that contribute to the acquisition of most caterpillar‐associated microbes (Hannula et al., 2019). Previous studies under field conditions in the M. *cinxia* suggest that the microbiome is highly variable and individuals collected within the same nest and consequently belonging to the same family growing on the same host plant on the same soil can harbour drastically different communities (Minard et al., 2019). However, this field study was conducted on closely related populations from the Sund region in Åland islands that use only *P. lanceolata* as a host plant. Our present study suggests that within this global variable microbial community, at least discrete members might be driven by factors intrinsic to the host population when those are more distant and experienced different histories of host plant usage.

#### 4.3.2 IGs induce only minor shifts in the larval microbiome

Interestingly, variation in the IG levels within the host plant the larvae was feeding on was only mildly correlated with the community composition of the larval microbiota. This suggests that those microbial communities are poorly affected by the antimicrobial activity of the IGs. Our results contrast with some previous findings conducted on the camellia weevil *Curculio chinensis*. The microbiota of larvae feeding on *Camellia* sp. seeds producing tea saponin are enriched in specific taxa when cultivated in a medium supplemented with this defence metabolite (Zhang et al., 2020). It was further shown that the bacteria were able to degrade tea saponins individually in vitro but that they were more efficient when present together as a community. Assuming that this was an important factor structuring the camellia weevil microbiota, results from our comparative study, shows that only a limited fraction of the microbiota in *M. cinxia* responded to a major class of defensive compounds, IGs, in one of its main host plants. In *M. cinxia*, changes in microbiota were only detected when assessments were made at the community but not at individual OTU level between the two treatments. This result is consistent with our previous field study showing that the composition of the bacterial microbiota in the larvae poorly correlates with the metabolome of the host plant (Minard et al., 2019). This result could either reflect (i) that the microbial community associated with the larvae is resistant to the IG compounds, (ii) that the antimicrobial activity of those compounds is attenuated after ingestion by the larvae or (iii) that the impact on the microbial communities is not dose‐dependent. In our study, the bacterial community at the surface of the leaves differs between plants producing high and low levels of iridoid glycosides. Furthermore, the bacterial and fungal communities differed substantially between plants and larvae. This suggests either that the larvae carry their own symbionts, which are not acquired from the host plant or that larvae select specific bacteria and fungi that might be rare at the plant surface. This is in line with previous studies showing that some insect herbivores do not acquire the majority of their microbiota from their host plant (Hannula et al., 2019) as well as with a field study of *M. cinxia* microbiota (Minard et al., 2019). Furthermore, several studies in Lepidoptera have suggested transient microbiota (rapidly excreted through the faeces after their acquisition) have only limited interactions with the host plant (Hammer et al., 2017, 2019). It is important to note, however, that the composition of the microbiota of lab reared larvae might differ from that of larvae observed in the field (Belda et al., 2011; Lighthart, 1988; Mason & Raffa, 2014; Xiang et al., 2006). Nonetheless, similar to a previous field experiment (Minard et al., 2019), larvae harboured a high abundance of Enterobacteriaceae. In comparison to this previous field study (conducted in diapausing larvae), the larval microbiota in both studies mainly consist of Enterobacteriaceae although they differ with respect to some other taxa (Minard et al., 2019). The plant microbiota seem to vary across the studies since some dominant Alphaproteobacteria (*Methylobacterium*, Aureimonas) retrieved in the field poorly colonized the plants in the laboratory.

#### 4.3.3 The parental origin in interaction with the presence of microbiota impact larval performance and their ability to cope with IGs

In our study, larvae from Eckerö, that encounter lower levels of IG in the field, had a better survival rate when fed with plants containing low IG levels and after antibiotic‐mediated microbiota disturbance. Furthermore, the larvae originating from Eckerö tended to be less affected by IG‐induced reduction of Lysin in that same condition. Conversely, no differences were observed in the larvae originating from Sund. This suggest that the larvae that are less often encountering host plants with high levels of IG may not be properly adapted to the host plant defences and that their microbiota mitigate such interactions. Interestingly, larvae originating from Sund region with only higher IG host plant present did exhibit low level of Lysin availability, but their survival or performance was not significantly affected by any of the treatments. These results may suggest that both the effect of the microbiota and host plant adaptation influence the response of larvae to plant defences.

## 5 CONCLUSIONS

The main defensive compounds are poorly stored in the larval tissues of early instar caterpillars, but the variation in the IG concentration of the host plant does impact the metabolism of this herbivore through a decrease of lysine in the insect tissues. This amino acid was also affected by the antibiotic treatment suggesting that the microbiota might also impact the metabolism of the larvae under certain conditions, possibly by interfering with the IG‐crosslinking to lysine. The development of the small larvae was slightly longer when they were fed with plants producing high levels of defensive compounds and their survival was slightly better when their microbiota were altered by antibiotic treatment. Finally, *M. cinxia* larvae harbour a microbiota that are poorly impacted by the region of origin or genetic background of the larvae and with the defensive compounds content of the host plant. Together, our results suggest that the metabolic cost of plant defence and microbiota might have mild but significant consequences for the performance of this specialist herbivore.

## AUTHOR CONTRIBUTIONS

Guillaume Minard and Marjo Saastamoinen conceived the ideas and designed the methodology. Guillaume Minard performed the experiments and data analysis. Aapo Kahilainen contributed to data analysis. Arjen Biere selected the plant lines. Hannu Pakkanen and Johanna Mappes contributed to the biochemical analysis. Guillaume Minard wrote the first draft version under the supervision of Marjo Saastamoinen. All authors contributed critically to the draft and approved its final version.

## CONFLICT OF INTEREST

Arjen Biere is an Associate Editor of *Functional Ecology*, but took no part in the peer review and decision‐making processes for this paper.

## Supporting information


Appendix S1

## Data Availability

Life‐history traits, microbiota and metabolomic data are available on Zenodo: https://zenodo.org/record/7045832#.YxdRyXbMI2w (Minard et al., 2022). Metabarcoding sequences have been submitted to the European Nucleotide Archive under the accession number (PRJEB53825 will be completed upon acceptance).
