# Supplementary material for: Complex plant quality—microbiota–population interactions modulate the response of a specialist herbivore to the defence of its host plant
Source: Funct Ecol. 2022 Sep 15;36(11):2873–88. doi: 10.1111/1365-2435.14177 (PMC9826300; doi:10.1111/1365-2435.14177)
Supplement: Supplementary file 1 — Appendix S1 [file FEC-36-2873-s001.docx]

**Complex plant quality - microbiota - population interactions modulate the response of a specialist herbivore to the defense of its host plant.**

Guillaume Minard, Aapo Kahilainen, Arjen Biere, Hannu Pakkanen, Johanna Mappes, Marjo Saastamoinen

**Supplementary methods**

*Plant lines selection*

A total of 10 genotypes from each selection Plant lines were grown in a greenhouse at the University of Helsinki in a mix of 50 % soil and 50 % perlite. All the seeds were planted simultaneously as the age of the plant is known to affect the level of defensive metabolites in *P. lanceolata* (Fuchs & Bowers, 2004)*.* The plants were grown under 18h day / 6h night conditions, were watered every 3 days and fertilized once a week. Among the 20 plant genotypes, 6 (3 High-IG and 3 Low-IG lines) were selected based on a metabolomics control of their IG production conducted 1 month before the experiment. A total of 3 young leaves per plant were harvested from 3 plants and from each of the 20 plant genotypes (except for the genotype H10 for which a too limited amount of seeds germinated) one month after they were grown. After cutting both ends of the leaves and freeze-drying the leftover parts, ^1^H-NMR metabolomics analysis were conducted on 10mg dried plant material following the protocol described in the core manuscript. Signals corresponding to iridoid glycosides (aucubin and catalpol) were normalized and analysed as well as verbascoside (Supplementary figure 1). Genotypes H2, H3 and H9 were selected as high-IG plant line representative. Genotypes L4, L6, L7 were selected as low-IG plant line representative. Rational for the selection was (i) a consistently high or low IG level and (ii) a sufficiently high germination rate in order to be able to harvest multiple plant individuals each days without harvesting the same plant two consecutive days. Between 14 and 21 individual plants were grown for each of the selected genotypes.

*Metabarcoding of the bacterial and fungal communities in the larval gut and the host plant*

Gut microbiota from three *M. cinxia* individuals per family per treatment were pooled. In addition, three 0.5 cm^2^ leaf pieces per plant genotype were analyzed. DNA was extracted from the larval gut samples or the host plant with Qiagen DNeasy Blood and Tissue kit (Qiagen, Germany) using an optimized protocol for extraction of bacterial DNA from low matrix (Minard *et al.,* 2015). For the estimation of the bacterial diversity, the V5-V6 hypervariable region of the 16S rDNA was amplified using the primers 784F (5’-AGGATTAGATACCCTGGTA-3’) and 1061R (5’-CRRCACGAGCTGACGAC-3’) modified by the addition of partial Illumina primers (Illumina, USA) (Andersson et al., 2008). The amplifications were carried out with a protocol of 5min at 95°C, 30 cycles including 40s at 95°C, 1min at 54.2°C, 30s at 72°C and a final extension step of 7min at 72°C. The PCR reaction mixture contained 1X of Reaction Buffer (New England Biolabs, USA), 1X of High GC enhancer (New England Biolabs, USA), 40µM of dNTP (Applied Biosystems, USA), 200nM of each primer, 8µg.µl^-1^ of Serum Bovine Albumine (new England Biolabs, USA), 1.2µg.µ^-1^ of T4gene32 (New England Biolabs. USA) and 15ng of DNA. A partial variable region of the 28S rDNA was amplified separately for non-Ascomycota fungi using the primers LSU200-F (5’-AACKGCGAGTGAAGMGGGA-3’) and LSU481-R (5’-TCTTTCCCTCACGGTACTTG-3’) as well as for Ascomycota fungi using the primers LSU200A-F (5’-AACKGCGAGTGAAGCRGYA-3’) and LSU476A-R (5’-CSATCACTSTACTTGTKCGC-3’) modified by the addition of partial Illumina adapters (Illumina, USA). Those two primer pairs were used for the fungi according to the previously published method (Asemaninejad et al., 2016) in order to cover the entire fungal diversity but only primers amplifying Ascomycetes succeeded to amplify fungi (presumably because of the poor abundance of other fungal taxa in the samples). PCR were performed with 15ng of DNA using a reaction mixture containing 1X of Phusion buffer (Thermo scientific, USA), 500nM of each primer, 200µM of dNTPs (Thermo scientific, USA), 3% of DMSO (Thermo scientific, USA) and 0.5 U of Phusion DNA polymerase (Thermo scientific, USA). Amplification steps were carried out with an initial denaturation at 98°C for 3min followed by 1 cycle of 20s at 98°C, 30s at 62°C (non-Ascomycota) or 55°C (Ascomycota), 18s at 72°C, 29 cycles of 20s at 98°C, 30s at 62°C, 18s at 72°C, and a final elongation step of 7min at 72°C. The final preparation of the samples and sequencing were processed by the Institute for Molecular Medicine Finland (FIMM) using the Miseq v.3. sequencing platform (Illumina, USA). In order to increase the sequence diversity, 5% of PhiX Phage DNA was added to the libraries prior to sequencing.

The sequences were analyzed with the pipeline mothur v.1.40.4 following the Standard Operating Procedure described by the developers (http://www.mothur.org/wiki/MiSeq_SOP) (Schloss et al., 2009). Sequences were deposited in the European Nucleotide Archive under the project number (PRJEB53825, will be available before acceptance)

*Bacterial and fungal quantification in the larval gut and the plant surface*

Bacterial and fungal densities in the larval midgut and plant genotypes described above were estimated based on qPCR measurements. The amplifications were conducted with the same primers as described above. qPCRs were run separately for bacterial 16S rDNA, fungal (Ascomycota) and fungal (non-Ascomycota) 28S rDNA. The reaction mix contained 1X of IQ SYBR Green supermix (Bio-Rad, USA) and 500nM of the primers. Amplifications were conducted with 3min of denaturation at 95°C and 40 Cycles of 10s of denaturation a 95°C and 60s at 55°C (16S rDNA) or 62°C (28S rDNA). Copy numbers of 16S rDNA and 28S rDNA were estimated based on a standard curve realized with serial dilutions of 10^8^ to 10^1^ copies of purified 16S rDNA and 28S rDNA amplicons of *Micrococcus luteus* (Eubacteria)*, Podosphaera plantaginis* (Ascomycota) and *Antrodia sitchensis* (Basidiomycota)*.* The community composition was corrected for the total abundance quantified for each primer by qPCR and mitochondrial or chloroplastic sequences were subtracted from those quantifications.

*^1^H-NMR metabolomics for plant and individual carcasses*

^1^H-NMR was used as it enables the simultaneous and reproducible quantification of Iridoid Glycosides (aucubin, catalpol), lysin and glucose, which are known to vary after IG activation by β-glucosidases, and verbascoside, which is a phenolic compound potentially involved in *P. lanceolata* herbivore defense. After cutting both ends of the leaves, the remaining tissue of leaf samples was crushed with a sterile pestle in liquid nitrogen and the frozen powder was freeze dried for 48h. A total of 3.5mg of dried powder was used for each plant sample. Larvae were dissected on ice and their digestive tract was removed as described above. Three individual larvae from each family in each treatment were pooled for metabolomics analysis. The larvae were freeze-dried for 48h and their dry mass was recorded. The extraction was processed using the previously described protocol (Kim *et al.,* 2010). Metabolites were extracted using a solvent containing 750 µl of CD_3_OD (VWR Chemicals, Belgium) and 750 µl of KH_2_PO_4_ (Sigma-Aldrich, Germany) buffer in D_2_O (pH6) with 0.05% (wt/wt) of TSP (sodium trimethylsilylpropionic acid) (Sigma-Aldrich, U.S.A.). The mixture was sonicated for 20min and then centrifuged at 17,000g for 10min. The supernatant was collected and 800 µl was transferred into a 5mm Ø NMR tube (Wilmad, U.S.A.). ^1^H-NMR spectra were acquired at 298K on a Bruker 850 MHz Avance III HD NMR spectrometer equipped with a TCI Cryoprobe (Bruker, U.S.A.). ^1^H NMR spectra were recorded using 1D presaturation pulse sequence (zgpr). For each ^1^H spectrum, 256 transients were collected into 32K time domain points using a 60° flip angle, spectral width of 10.2 kHz, relaxation delay of 5.0s, an acquisition time of 1.6s, and a mixing time of 5ms. Fourier transformation of the free-induction decay was applied with zero filling to give 65K frequency domain data points. Prior to Fourier transformation, 0.3-line broadening was applied. ^1^H chemical shifts were referenced to an internal standard TSP at δ 0.0 ppm. NMR spectra were processed with MNOVA software v.10.0.2 (Mestrelab research S.L., Spain). Model compounds of aucubin (Sigma-Aldrich, Germany), catalpol (Sigma-Aldrich, Germany) and verbascoside (Extrasynthese, France) were used for signal assignments of *P. lanceolata* defensive metabolites. Other primary or secondary metabolite shifts and J-coupling constants obtained from plant material using similar solvents were used as a reference (Kim *et al.,* 2010; Lubbe *et al.,* 2011; Yang *et al.,* 2012; Agudelo-Romero *et al.,* 2014; Gallo *et al.,* 2014). For multivariate analysis, the signal was binned to 0.04 ppm and integrated. The TSP and CD_3_OD signals were removed and the integral values were transformed following the given formula:

$$\frac{9\times\int_{\delta-0.02}^{\delta+0.02} Intensity}{DW\times\int_{-0.02}^{0.02} Intensity of TSP}$$

Where *DW* represents the exact dry weight of the sample (±0.1mg), *δ* represents the ^1^H chemical shift and 9 refers to the number of equivalent ^1^H atoms contained within the TSP reference molecule.

*LC-ESI-MS quantification of Iridoid Glycosides within larval carcasses*

Since ^1^H-NMR was not sensitive enough to detect and efficiently quantify the variations of aucubin and catalpol within the larval carcasses, extractions from carcasses were analysed by a liquid chromatograph equipped with electrospray tandem mass spectrometer (LC-ESI-MS). Carcasses from three individuals per treatment group were pooled together in a 1.5 ml tube after removal of the digestive tract from each larva. Pooled samples were frozen in liquid nitrogen, freeze-dried and weighed.

Aucubin and catalpol (both Rotichrom HPLC quality) were purchased from Roth. HPLC grade methanol and acetonitrile were supplied by J.T. Baker. Ammonium acetate (puriss. p.a.) was provided by Riedel-de Haën. Water was prepared with a Milli-Q water purification system from Millipore. Ground material was extracted in 1 ml 7 % methanol. The samples were first vortex-mixed and let stand overnight. The crude extract was filtered through 0.45 µm PVDF syringe filter and then measured with LC-ESI-MS. Standard stock solutions of aucubin and catalpol (100 µg/mL) were prepared in 50% methanol. Working solutions of lower concentration were prepared by appropriate dilution of the stock solution.

The LC-ESI-MS/MS/MS system consists of an Agilent 1290 ultra-high pressure LC system and an Agilent 6460 QQQ mass spectrometer (Agilent Technol. Inc., Palo Alto, CA USA). Separation was performed on a Zorbax Eclipse Plus C18 column (100 mm × 2.1 mm, 1.8 µm; Agilent, USA) protected by a Zorbax SB-C18 UHPLC Guard (2.1 mm × 5 mm, 1.8 µm). The temperature of column was set at 30 °C and the volume of sample injection was 3 µL. The gradient elution, with mobile phases of 5 mM ammonium acetate in water (A) and acetonitrile (B), was carried out by employing at a total ﬂow rate of 0.3 mL/min (**Supplementary Table 2**).


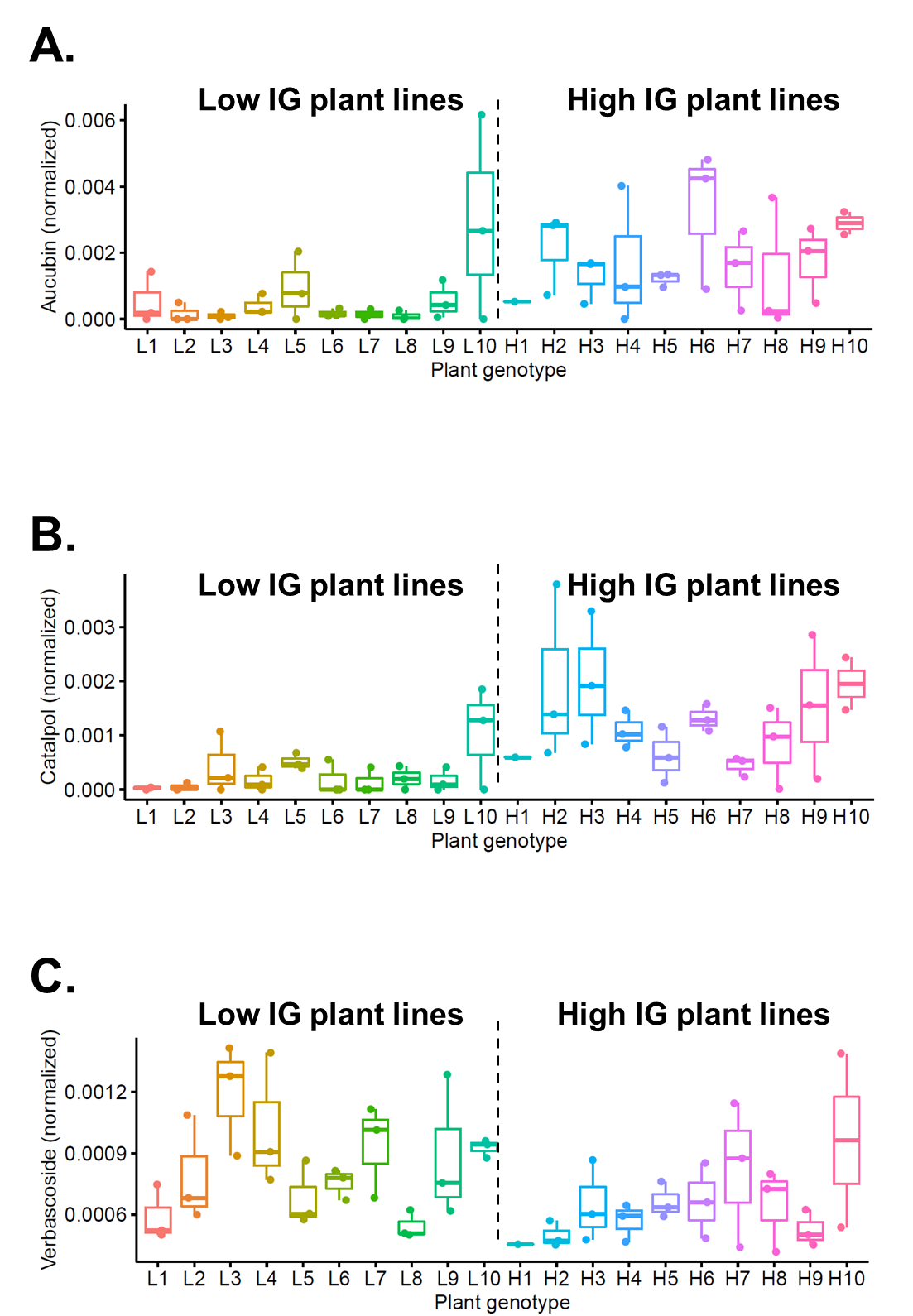


**Supplementary figure 1. Defensive metabolite levels in plant genotypes.** Plants were selected to produce either high or low-IG. The defensive metabolites were measured with ^1^H-NMR and integrals were normalized. Each of the two IGs (A) Aucubin and (B) Catalpol were reported as well as (C) Verbascoside : another defensive metabolite of *Plantago lanceolata.*

**
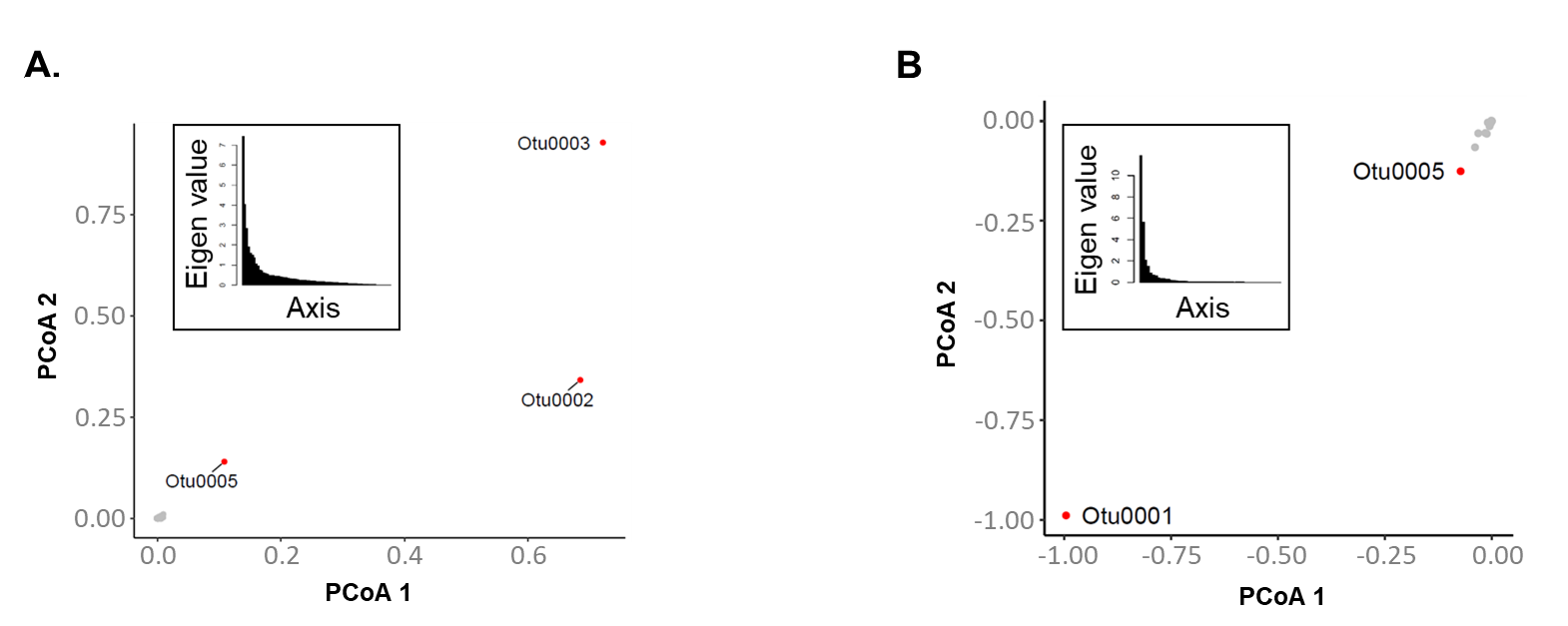
**

**Supplementary figure 2. OTUs inducing major variations in the microbial communities associated with *M. cinxia* larvae across all conditions.** OTUs loadings based on PCoA analysis of the Bray-Curtis distances are represented (A) for the bacteria and (B) for the fungi. The OTUs that differed mostly for the centroid are represented in red while others are represented in grey.


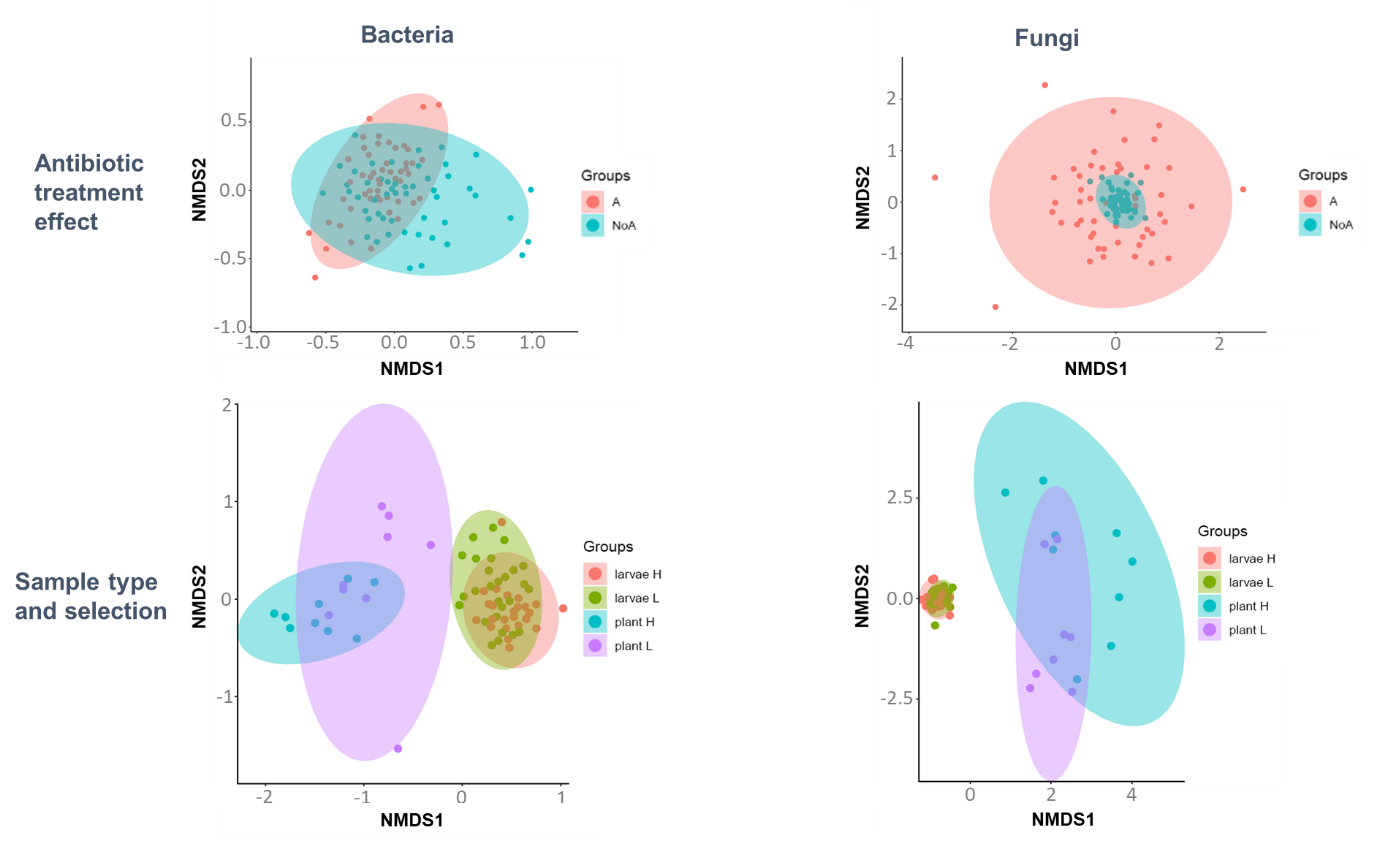


**Supplementary figure 3. Variation in the microbial community composition in response to antibiotic treatment, plant selection and sample type.** The community similarities were represented based on a Non-metric multidimensional scaling of the Bray-Curtis dissimilarity distances for the bacterial and fungal communities. Comparisons of antibiotic treated (A) and non treated (NoA) individuals were performed as well and comparisons of plant and larvae samples between high-IG (H) and low-IG (L) lines.

**
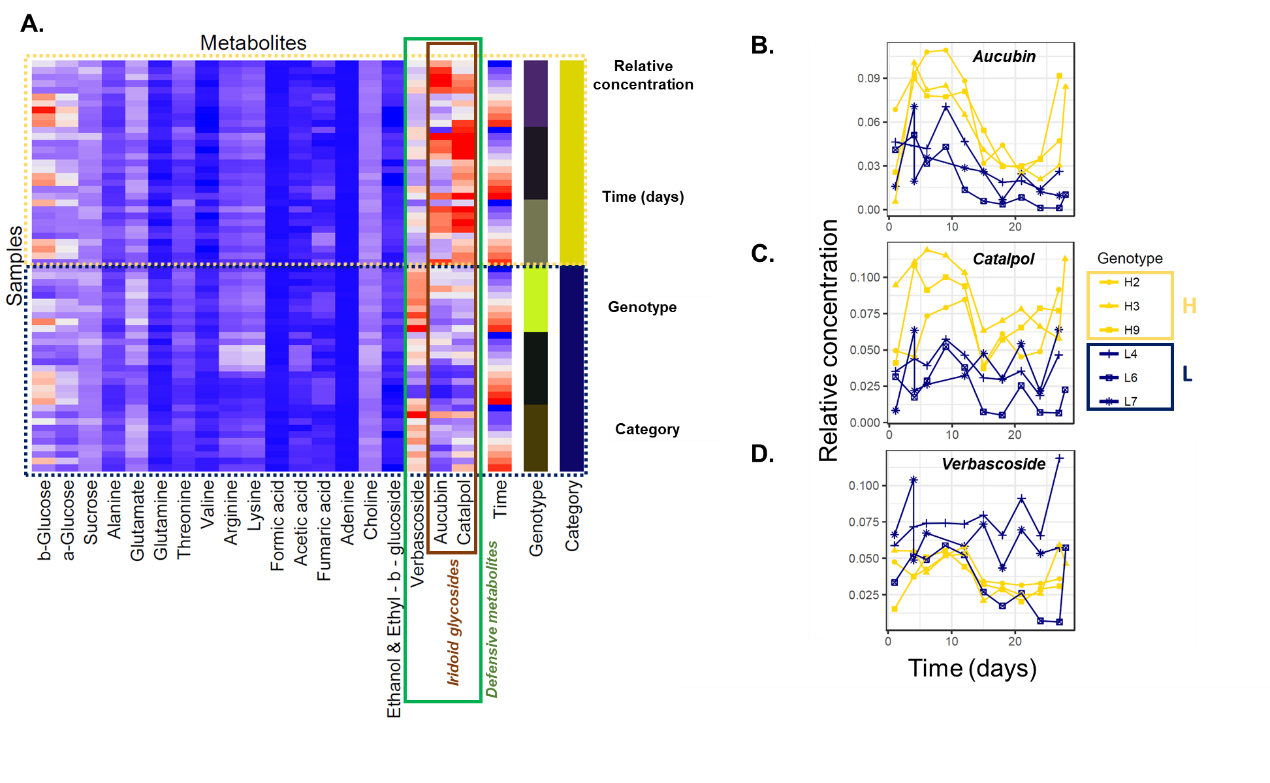
**

**Supplementary figure 4. Control of the metabolome of the host plant.** The concentration relative to the mass of the plant leaves and the integral value of the TSP internal standard of (A) each annotated metabolites measured with 1H-NMR are reported for each plant selection line (L = low production of IGs, H = high production of IGs), each plant genotype within a selection line, and from the beginning (Time = 1 day) until the end (Time = 27 days) of the experiment. A specific focus was performed for the three main defensive metabolites namely the two IGs (B) Aucubin, (C) Catalpol and the phenolic compound (D) Verbascoside.

**
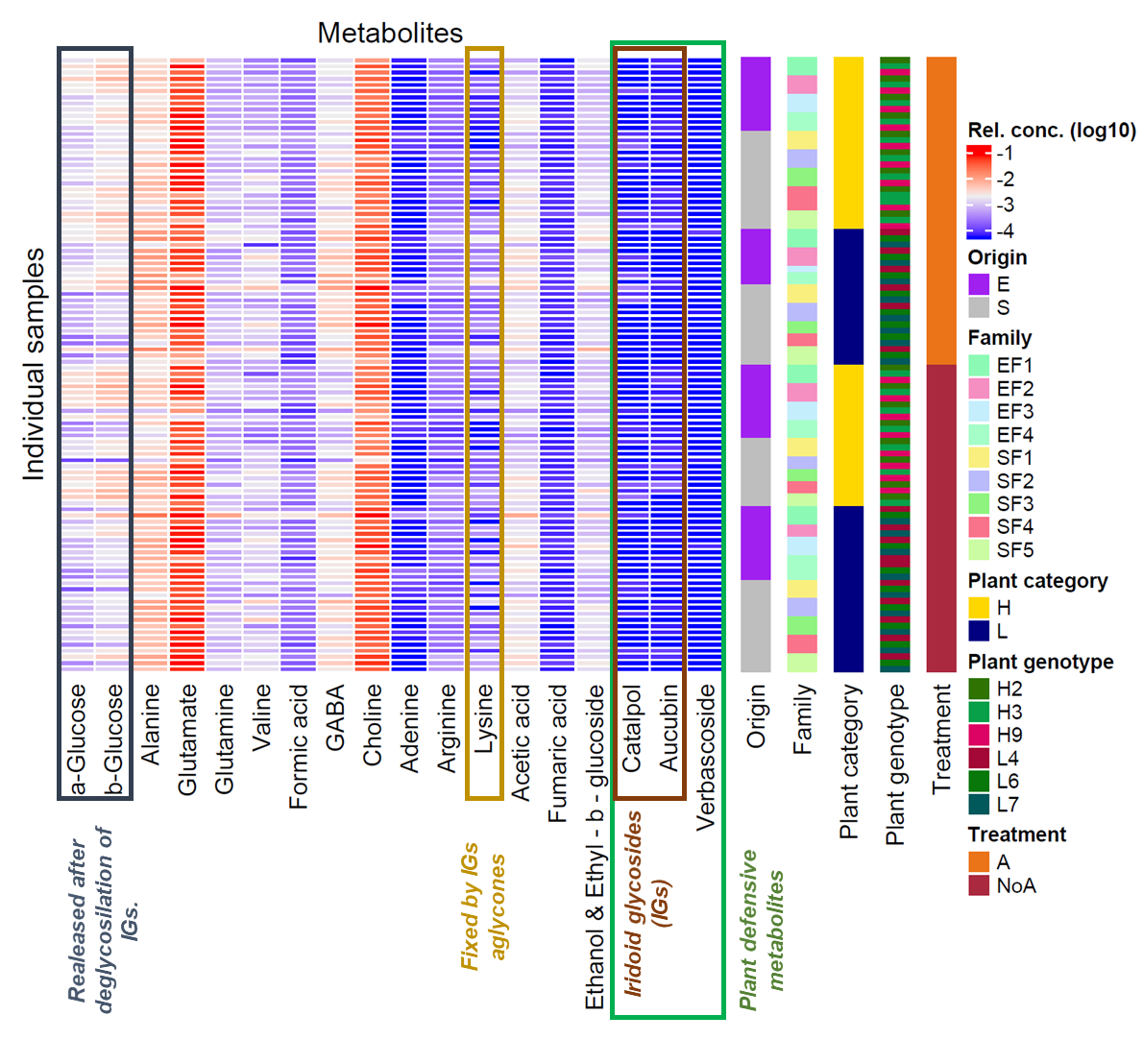
**

**Supplementary figure 5. Impact of the plant metabolite content and the antibiotic treatment on the larval metabolome characterized with ^1^H-NMR.** The relative concentration (log10 transformed) of each annotated metabolite measured with 1H-NMR in the carcasses of samples fed with various plant genotypes belonging to a given plant selection line (L = low production of IGs, H = high production of IGs), their larval family number, and larval origin are reported (E = Eckerö or S = Sund) as well as the larval treatment (A = antibiotic treated, NoA = no treatment).

**
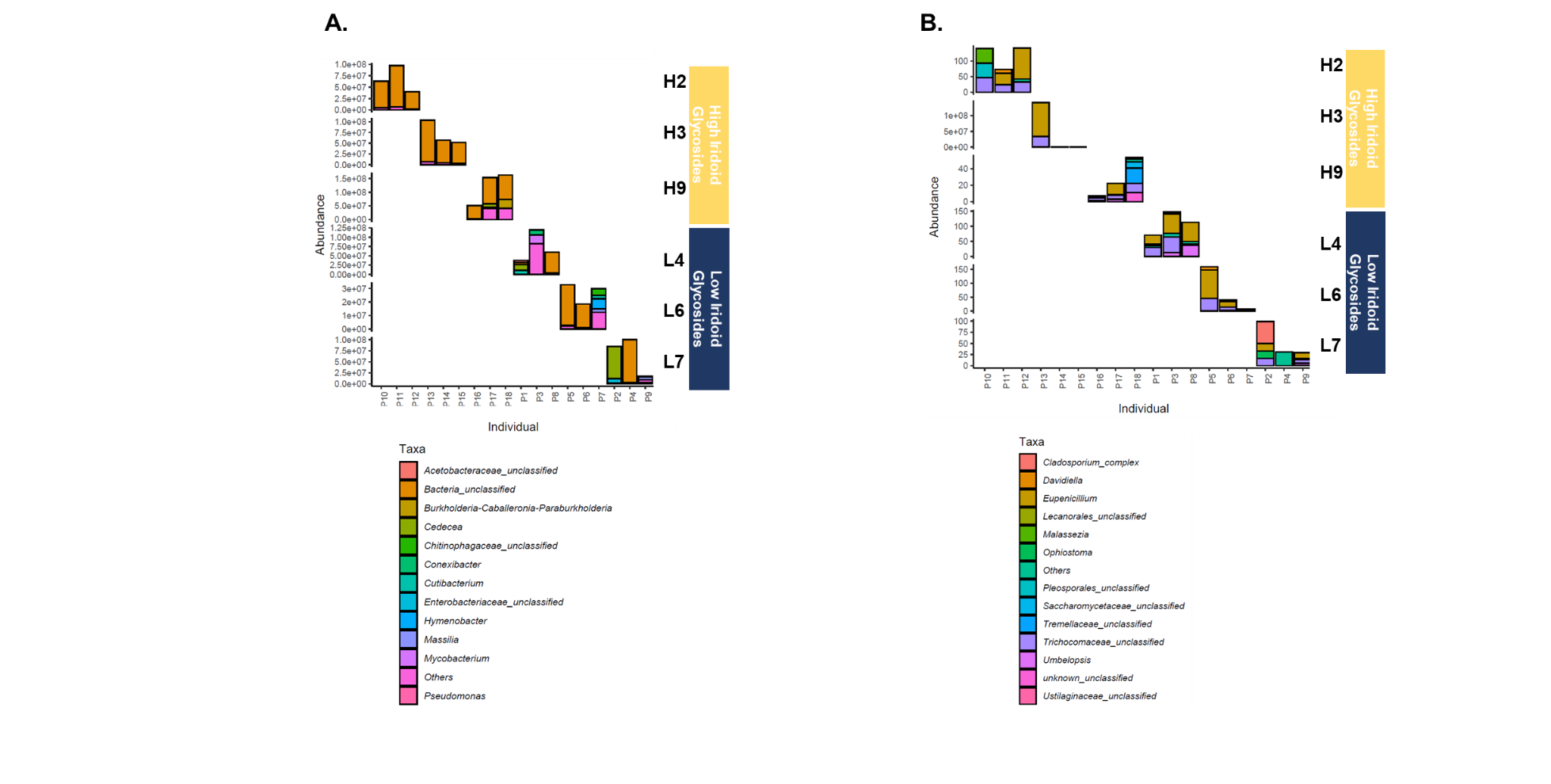
**

**Supplementary figure 6. Composition of the plant microbiota.** Abundance of the different (A) bacterial or (B) fungal taxa retrieved in plants selected to produce High or Low-IG are represented for each plant genotype (H2, H3, H9, L4, L6, L7). Three pools of plant leaves (referred here as ‘Individual’) were analyzed.

**
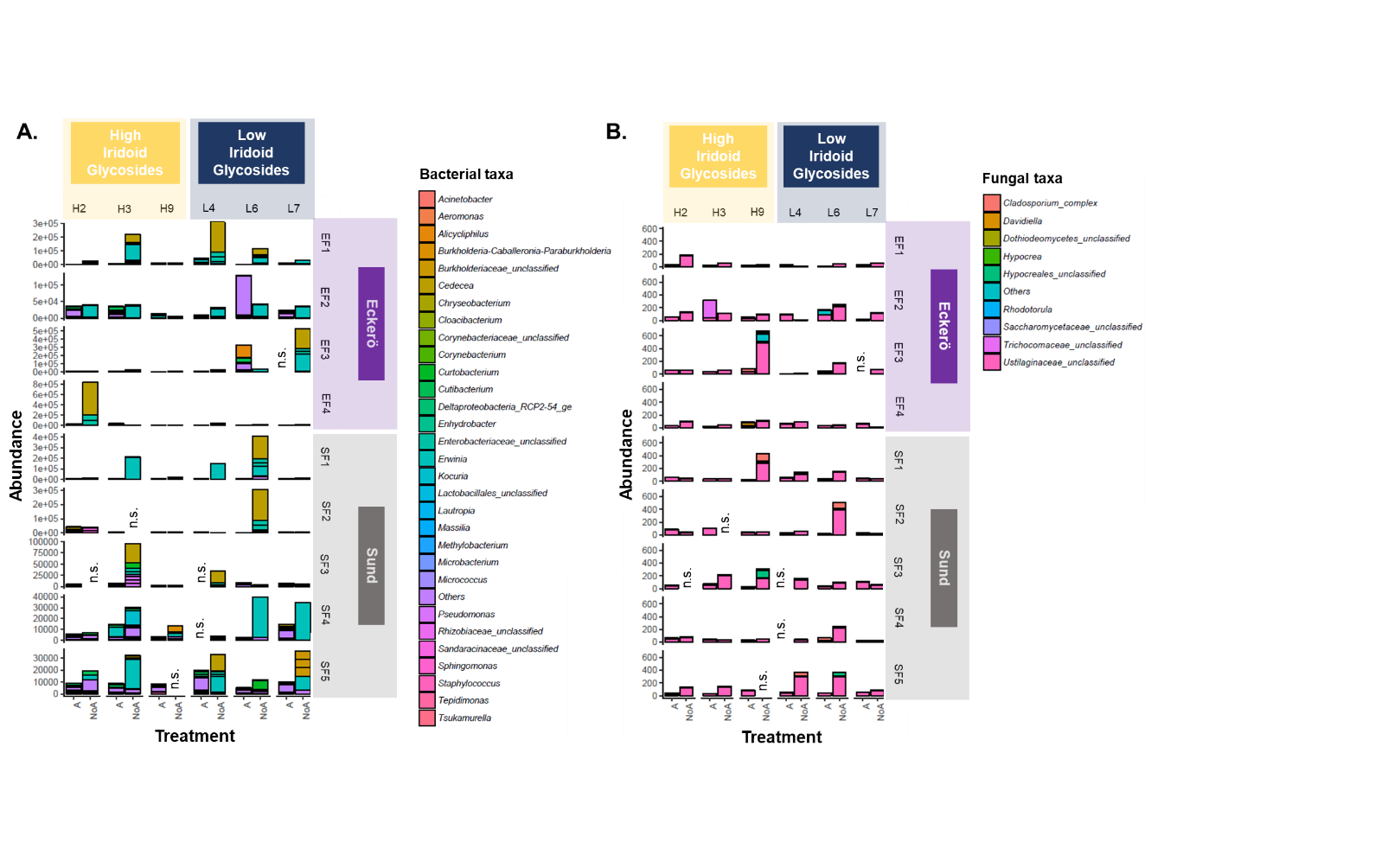
**

**Supplementary figure 7. Composition of the microbiota across the treatment groups.** The composition of (A) bacterial and (B) fungal communities have been reported for each family derived from parents collected in Eckerö and Sund fed with plant genotypes selected to produce either either high (H2, H3, H9) or low (L4, L6, L7) levels of IGs whether they were treated or not (A and NoA) with antibiotics. The relative abundances were corrected by the absolute abundance of each group estimated by qPCR of the 16s (bacteria) and the 28S (fungi) rRNA genes. n.s. represent groups of individuals for which the number of surviving individuals was not sufficient to allow the measurement.

**Supplementary table 1. Gradient elution used in LC-ESI-MS runs**

| t (min) | (A) 5 mM ammonium acetate–water (%) | (B) Acetonitrile (%) |
| --- | --- | --- |
| 0.0 | 94 | 6 |
| 0.2 | 94 | 6 |
| 5 | 80 | 20 |
| 6 | 50 | 50 |
| 7 | 50 | 50 |
| 7.5 | 94 | 6 |
| 7.9 | 94 | 6 |

| **Supplementary table 2. Factors influencing the abundance of the main bacterial and fungal taxa.** | | | | | |
| --- | --- | --- | --- | --- | --- |
| Community | Variable | Factor | df. | F | p-value |
| Bacteria | Otu0002\| Cedecea | Plant lines | 1,4.004 | 1.2506 | 0.326 |
|  |  | Treatment | 1,82.900 | 45.4543 | **1.942x10^-9^***** |
|  |  | Origin | 1,6.825 | 2.8615 | 0.136 |
|  |  | Plant lines x Treatment | 1,82.844 | 2.6536 | 0.107 |
|  |  | Plant lines x Origin | 1,83.070 | 0.1541 | 0.696 |
|  |  | Treatment x Origin | 1,83.070 | 5.9634 | **0.016*** |
|  |  | Plant lines x Treatment x Origin | 1,83.010 | 0.2428 | 0.624 |
|  | Otu0005\| Enterobacteriaceae unclassified | Plant lines | 1,4.036 | 3.2237 | 0.146 |
|  |  | Treatment | 1,83.034 | 16.0144 | **0.0001***** |
|  |  | Origin | 1,7.011 | 0.0259 | 0.877 |
|  |  | Plant lines x Treatment | 1,83.001 | 0.0426 | 0.837 |
|  |  | Plant lines x Origin | 1,83.354 | 1.4687 | 0.229 |
|  |  | Treatment x Origin | 1,83.354 | 0.6920 | 0.408 |
|  |  | Plant lines x Treatment x Origin | 1,83.316 | 0.0332 | 0.856 |
|  | Otu0001\| Ustilaginaceae unclassified | Plant lines | 1,87.279 | 0.591 | 0.444 |
|  |  | Treatment | 1,87.280 | 19.2836 | **3.15x10^-5^***** |
|  |  | Origin | 1,7.043 | 0.121 | 0.737 |
|  |  | Plant lines x Treatment | 1,87.237 | 0.5075 | 0.478 |
|  |  | Plant lines x Origin | 1,87.280 | 0.1093 | 0.742 |
|  |  | Treatment x Origin | 1,87.280 | 2.4578 | 0.121 |
|  |  | Plant lines x Treatment x Origin |  |  |  |
| Fungi | Otu0001\| Ustilaginaceae unclassified | Plant lines | 1,3.941 | 0.8973 | 0.398 |
|  |  | Treatment | 1,83.357 | 133.3307 | **<2x10^-16^***** |
|  |  | Origin | 1,7.081 | 0.2093 | 0.661 |
|  |  | Plant lines x Treatment | 1,83.282 | 0.3219 | 0.572 |
|  |  | Plant lines x Origin | 1,83.612 | 0.6400 | 0.426 |
|  |  | Treatment x Origin | 1,83.612 | 1.4263 | 0.236 |
|  |  | Plant lines x Treatment x Origin | 1,83.534 | 1.9271 | 0.169 |
|  | Otu0005\| Cladosporium complex | Plant lines | 1,87.749 | 0.5437 | 0.463 |
|  |  | Treatment | 1,87.749 | 14.0836 | **0.0003***** |
|  |  | Origin | 1,7.225 | 2.0562 | 0.193 |
|  |  | Plant lines x Treatment | 1,87.655 | 0.1885 | 0.665 |
|  |  | Plant lines x Origin | 1,87.749 | 0.2274 | 0.635 |
|  |  | Treatment x Origin | 1,87.749 | 7.6145 | **0.007**** |
|  |  | Plant lines x Treatment x Origin | 1,87.655 | 0.0534 | 0.818 |
| p-value : .≤0.1, *≤0.05, **≤0.01, ***≤0.001 | |  |  |  |  |

| **Supplementary table 3. Factors influencing the variations of the plant microbiota** | | | | | |
| --- | --- | --- | --- | --- | --- |
|  | Factors | Df. | pseudo F | R^2^ | p-value |
| **Bacteria** |  |  |  |  |  |
|  | Plant lines | 1 | 3.516 | 0.184 | **0.005*** |
|  | Plant lines x Genotype | 4 | 0.897 | 0.188 | 0.615 |
|  | Residuals | 12 |  | 0.628 |  |
| **Fungi** |  |  |  |  |  |
|  | Plant lines | 1 | 0.921 | 0.054 | 0.519 |
|  | Plant lines x Genotype | 4 | 1.003 | 0.237 | 0.467 |
|  | Residuals | 12 |  |  |  |
| p-value : .≤0.1, *≤0.05, **≤0.01, ***≤0.001 | | |  |  |  |

**References**

Andersson, A. F., Lindberg, M., Jakobsson, H., Bäckhed, F., Nyrén, P., & Engstrand, L. (2008). Comparative Analysis of Human Gut Microbiota by Barcoded Pyrosequencing. *PLoS ONE*, *3*(7), e2836. https://doi.org/10.1371/journal.pone.0002836

Asemaninejad, A., Weerasuriya, N., Gloor, G. B., Lindo, Z., & Thorn, R. G. (2016). New Primers for Discovering Fungal Diversity Using Nuclear Large Ribosomal DNA. *PLoS ONE*, *11*(7). https://doi.org/10.1371/journal.pone.0159043

Bates, D., Maechler, M., Bolker, B., Walker, S., Christensen, R. H. B., Singmann, H., Dai, B., Grothendieck, G., & Green, P. (2017). *lme4: Linear Mixed-Effects Models using “Eigen” and S4* (1.1-13) [Computer software]. https://cran.r-project.org/web/packages/lme4/index.html

Chung, S. H., Rosa, C., Scully, E. D., Peiffer, M., Tooker, J. F., Hoover, K., Luthe, D. S., & Felton, G. W. (2013). Herbivore exploits orally secreted bacteria to suppress plant defenses. *Proceedings of the National Academy of Sciences*, *110*(39), 15728–15733. https://doi.org/10.1073/pnas.1308867110

Fuchs, A., & Bowers, M. D. (2004). Patterns of iridoid glycoside production and induction in Plantago lanceolata and the importance of plant age. *Journal of Chemical Ecology*, *30*(9), 1723–1741.

Hanski, I., Schulz, T., Wong, S. C., Ahola, V., Ruokolainen, A., & Ojanen, S. P. (2017). Ecological and genetic basis of metapopulation persistence of the Glanville fritillary butterfly in fragmented landscapes. *Nature Communications*, *8*. https://doi.org/10.1038/ncomms14504

Kuussaari, M., Singer, M., & Hanski, I. (2000). Local Specialization and Landscape-Level Influence on Host Use in an Herbivorous Insect. *Ecology*, *81*(8), 2177–2187. https://doi.org/10.1890/0012-9658(2000)081[2177:LSALLI]2.0.CO;2

Lenth, R. V., Buerkner, P., Herve, M., Love, J., Miguez, F., Riebl, H., & Singmann, H. (2022). *emmeans: Estimated Marginal Means, aka Least-Squares Means* (1.7.4-1) [Computer software]. https://CRAN.R-project.org/package=emmeans

Marak, H. B., Biere, A., & Damme, J. M. M. V. (2000). Direct and correlated responses to selection on iridoid glycosides in Plantago lanceolata L. *Journal of Evolutionary Biology*, *13*(6), 985–996. https://doi.org/10.1046/j.1420-9101.2000.00233.x

Nair, A., Fountain, T., Ikonen, S., Ojanen, P. S., & van Nouhuys, S. (2016). Spatial and temporal genetic structure at the fourth trophic level in a fragmented landscape. *Proceedings of Royal Society B: Biological Sciences, DOI: 10.1098/Rspb.2016.0668*, *283*(1831). http://rspb.royalsocietypublishing.org/content/283/1831/20160668.abstract

Oksanen, J., Blanchet, F. G., Kindt, R., Legendre, P., Minchin, P. R., O’Hara, R. B., Simpson, G. L., Solymos, P., Stevens, M. H. H., & Wagner, H. (2013). *vegan: Community Ecology Package* (2.0-10) [Computer software]. http://cran.r-project.org/web/packages/vegan/index.html

R Core Team. (2016). *R: A Language and Environment for Statistical Computing*. https://www.R-project.org/

Saastamoinen, M. (2007). Life-history, genotypic, and environmental correlates of clutch size in the Glanville fritillary butterfly. *Ecological Entomology*, *32*(2), 235–242. https://doi.org/10.1111/j.1365-2311.2007.00865.x

Saastamoinen, M., van Nouhuys, S., Nieminen, M., O’Hara, B., & Suomi, J. (2007). Development and survival of a specialist herbivore, Melitaea cinxia, on host plants producing high and low concentrations of iridoid glycosides. *Annales Zoologici Fennici*, *44*(1), 70–80. JSTOR.

Schloss, P. D., Westcott, S. L., Ryabin, T., Hall, J. R., Hartmann, M., Hollister, E. B., Lesniewski, R. A., Oakley, B. B., Parks, D. H., Robinson, C. J., Sahl, J. W., Stres, B., Thallinger, G. G., Van Horn, D. J., & Weber, C. F. (2009). Introducing mothur: Open-source, platform-independent, community-supported software for describing and comparing microbial communities. *Applied and Environmental Microbiology*, *75*(23), 7537–7541. https://doi.org/10.1128/AEM.01541-09
